# Skeletal Muscle Mass Modifies the Prognostic Impact of LDL Cholesterol in Chronic Heart Failure

**DOI:** 10.1002/jcsm.70168

**Published:** 2026-01-16

**Authors:** Ryosuke Sato, Tania Garfias‐Veitl, Guglielmo Fibbi, Mirela Vatic, Wolfram Doehner, Stefan D. Anker, Stephan von Haehling

**Affiliations:** ^1^ Department of Cardiology and Pneumology University Medical Center Göttingen Göttingen Germany; ^2^ DZHK (German Center for Cardiovascular Research), Partner Site Lower Saxony Göttingen Germany; ^3^ Department of Medical Statistics University Medical Center Göttingen Göttingen Germany; ^4^ Department of Geriatrics University Medical Center Göttingen Göttingen Germany; ^5^ Berlin Institute of Health Center for Regenerative Therapies (BCRT) Charité—Universitätsmedizin Berlin Berlin Germany; ^6^ Department of Cardiology (Virchow‐Klinikum) Charité University Medical Center Berlin Berlin Germany; ^7^ German Centre for Cardiovascular Research (DZHK) partner site Berlin, Charité Universitätsmedizin Berlin Germany; ^8^ Department of Cardiology (CVK) of German Heart Center Charité Berlin Germany

**Keywords:** cholesterol paradox, heart failure, low‐density lipoprotein cholesterol, mortality, skeletal muscle mass

## Abstract

**Background:**

Dyslipidaemia is among the major risk factors for atherosclerotic cardiovascular disease. Paradoxically, higher cholesterol levels are associated with better survival in heart failure (HF) of any aetiology. Because cholesterol is an integral component of skeletal muscle structure, one possible explanation involves the interplay between lipid metabolism and skeletal muscle health. Using data from the Studies Investigating Comorbidities Aggravating Heart Failure, we investigated whether an association exists between low‐density lipoprotein cholesterol (LDL‐C) levels and all‐cause mortality in patients with chronic HF in the context of skeletal muscle mass.

**Methods:**

A total of 241 patients with chronic HF (68 ± 11 years, 80% male, left ventricular ejection fraction 39% ± 13%) were enrolled. LDL‐C levels were divided into low and high based on the median value (93 mg/dL). The appendicular skeletal muscle mass index (ASMI) was assessed using a dual‐energy X‐ray absorptiometry scan and divided into high and low based on the sex‐specific median value (men = 7.97 kg/m^2^, women = 6.87 kg/m^2^). Patients were divided into four groups based on median LDL‐C levels and ASMI values.

**Results:**

During a median follow‐up of 6.3 years, 95 patients (39%) died. There were differences in mortality between the four groups, with the highest mortality in patients with low LDL‐C levels and low ASMI and the lowest mortality in patients with high LDL‐C levels and high ASMI (*p* = 0.002, by log‐rank). The low LDL‐C group had higher mortality compared with the high LDL‐C group (46% vs. 33%, *p* = 0.01, by log‐rank). Multivariate Cox proportional hazard analysis confirmed the association between the low LDL‐C group and higher mortality (adjusted hazard ratio [aHR] 1.65 [1.00–2.72], *p* = 0.04). This prognostic impact of low LDL‐C appeared greater in the low ASMI group than in the high ASMI group (56% vs. 37%, *p* = 0.01, and 36% vs. 28%, *p* = 0.28, by log‐rank: *p* for interaction = 0.47). In the low ASMI group, lower LDL‐C levels were predictors of mortality in multivariate Cox proportional hazard models (aHR 1.46–1.48 per 1 SD decrease in LDL‐C, all *p* < 0.05).

**Conclusions:**

In patients with chronic HF, lower LDL‐C levels were associated with higher mortality, especially in the low ASMI group. These findings suggest that low cholesterol levels may exacerbate skeletal muscle loss, potentially creating a vicious cycle that worsens patient outcomes. Lipid‐modulating strategies could help mitigate muscle wasting in HF.

## 1 Introduction

Dyslipidaemia is one of the major risk factors for atherosclerotic cardiovascular disease (ASCVD), with a global prevalence estimated to be as high as 40% [1]. A substantial body of evidence has demonstrated the effectiveness of intensive low‐density lipoprotein cholesterol (LDL‐C) lowering therapy with statins and proprotein convertase subtilisin‐kexin type 9 (PCSK‐9) inhibitors, and the concept of ‘the lower, the better’ has been well established for both primary and secondary prevention in ASCVD [2, 3, 4]. In contrast, several observational studies have shown that lower LDL‐C levels are associated with the development of diabetes and atrial fibrillation, as well as adverse clinical outcomes in patients with coronary artery disease (CAD), a paradoxical phenomenon referred to as the ‘cholesterol paradox’ [5, 6, 7]. Similarly, an inverse association between lower cholesterol levels and worse survival has been reported in patients with heart failure (HF) [8, 9]. The effects of statin therapy in patients with HF also remain uncertain, with inconsistent findings across studies [10, 11]. On this basis, recent HF guidelines do not recommend routine lipid‐lowering therapy without clear indications such as CAD [12, 13].

Understanding this situation is important because the lack of sufficient amounts of cholesterol may have detrimental effects in HF. Indeed, cholesterol is an integral component of skeletal muscle structure, which is, for example, involved in excitation–contraction coupling and glucose transport, helping to explain the interplay between lipid metabolism and skeletal muscle health [14, 15]. Additionally, lower cholesterol levels serve as markers of malnutrition, chronic inflammation, impaired immune function and diminished metabolic reserve [7, 8, 16], all of which are intimately linked to muscle wasting. Reduced muscle mass or function has already been linked to worse clinical outcomes in patients with HF by various groups of researchers, including our own [17, 18, 19, 20]. Nevertheless, previous studies have not systematically examined whether skeletal muscle mass modifies the association between LDL‐C levels and prognosis in patients with HF.

Using data from the Studies Investigating Comorbidities Aggravating Heart Failure (SICA‐HF), the present study aims to investigate the association between LDL‐C levels and all‐cause mortality in patients with chronic HF in the context of skeletal muscle mass [21, 22].

## 2 Methods

### 2.1 Study Population

**FIGURE 1:**
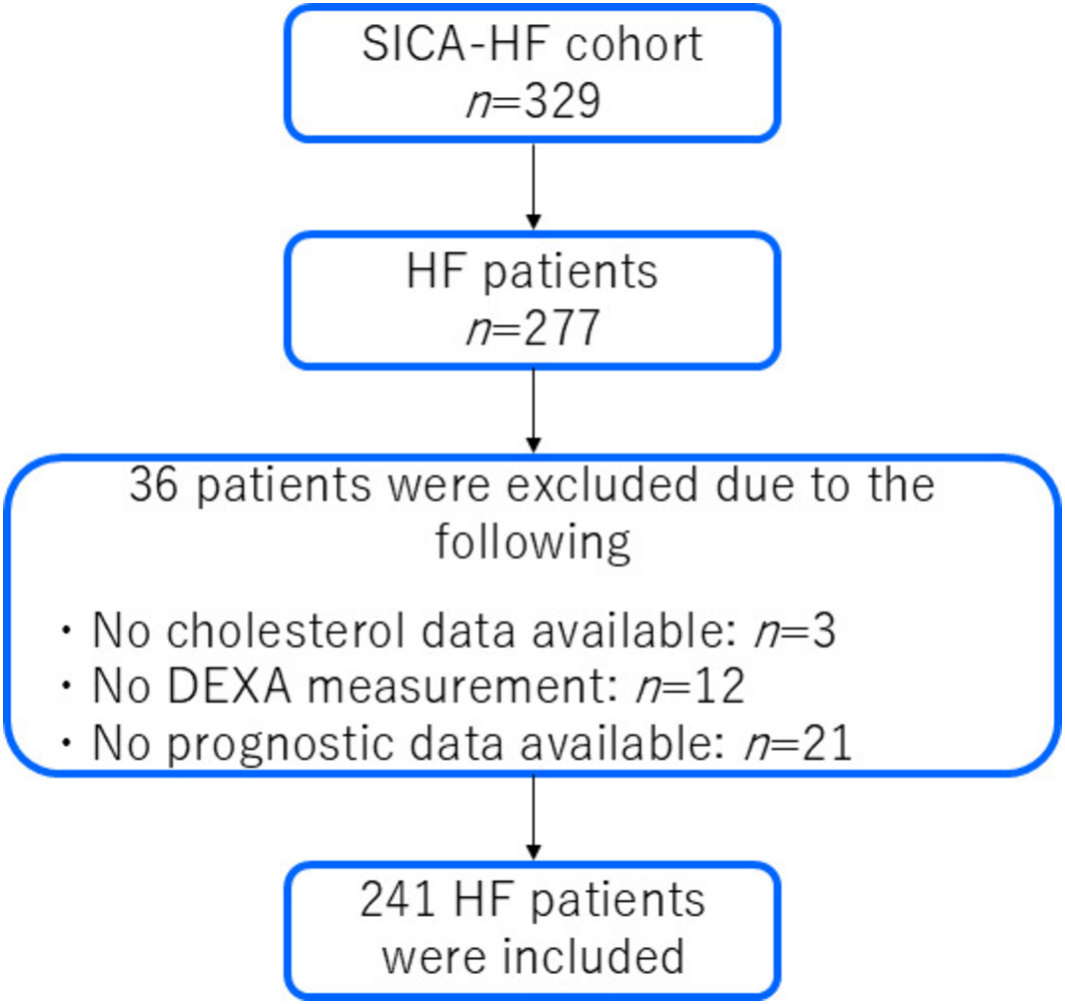
Study flow chart. DEXA, dual‐energy X‐ray absorptiometry; HF, heart failure, SICA‐HF, Studies Investigating Comorbidities Aggravating Heart Failure.

From March 2010 to April 2012, we enrolled 329 subjects in the SICA‐HF project at Charité—Universitätsmedizin Berlin, Campus Virchow‐Klinikum, Germany. After excluding controls and subjects without survival data, lipid profiles or body composition measurements, a total of 241 ambulatory HF patients with complete data (survival data, lipid profiles and dual‐energy X‐ray absorptiometry [DEXA] scans) were retrospectively analysed (Figure 1). The details and first results of SICA‐HF have been published previously [21, 22]. Briefly, clinically stable ambulatory chronic HF patients aged 18 years or above with clinical signs and symptoms of chronic HF were eligible for participation if at least one of the following criteria was satisfied: (1) left ventricular ejection fraction (LVEF) ≤ 40%, (2) left atrial dimensions > 4.0 cm (> 2.5 cm/m height) and (3) N‐terminal pro‐B‐type natriuretic peptide (NT‐proBNP) > 400 pg/mL or BNP > 150 pg/mL. The exclusion criteria were as follows: previous heart transplantation, a history of unstable angina, myocardial infarction, stroke, cardiovascular revascularization and open abdominal surgery within 6 weeks prior to the planned baseline visit. Patients with pregnancy or those on haemodialysis at baseline were also excluded. All subjects provided written informed consent at enrolment, and the local ethics committees approved the protocol. The study was funded by the European Commission's 7th Framework Programme (FP7/2007–2013) under grant agreement number 241558 and fulfilled all principles of the Declaration of Helsinki. SICA‐HF is registered under the ClinicalTrials.gov identifier: NCT01872299.

### 2.2 Assessment of Muscle Mass, Fat Mass, Muscle Strength and Exercise Capacity

The appendicular skeletal muscle mass and total body fat mass were evaluated using DEXA, with data analysis performed using a scanner model Lunar Prodigy and Lunar en Core 2002 software (GE Medical Systems, Madison, WI, USA). Appendicular skeletal muscle mass index (ASMI) was calculated as the sum of both arms and legs' lean soft tissue mass divided by height squared (kg/m^2^) [23], and patients were divided into low and high ASMI based on the sex‐specific median (men = 7.97 kg/m^2^, women = 6.87 kg/m^2^). Similarly, total body fat mass was divided by height squared (kg/m^2^). Muscle wasting was defined according to previously published criteria, that is, an appendicular skeletal muscle mass two standard deviations below the mean of a healthy young reference group aged 18–40 years (men < 7.26 kg/m^2^, women < 5.45 kg/m^2^) [23]. Cardiac cachexia was defined as the presence of nonoedematous, nonintentional weight loss of ≥ 5% over a period of at least 1 year [24]. Handgrip and quadriceps strength were measured using handgrip dynamometers (Saehan Corporation Korea Hydraulic Hand Dynamometer, model SH5001) and isokinetic dynamometers (Multitrace 2, Lectromed, Jersey, Channel Islands), respectively, and the best of the three measurements was used in both cases. Exercise capacity was assessed by a 6‐min walk test, a Short Physical Performance Battery (SPPB) [25] and a treadmill cardiopulmonary exercise testing with a modified Bruce protocol for peak oxygen uptake (VO_2_) [26] (a Naughton protocol for some patients) [27], all with standard protocols [28].

### 2.3 Laboratory Analysis

Blood samples were collected from an antecubital vein in the early morning following overnight fasting and at least 15 min of rest in the supine position. Blood cell counts and standard clinical biochemical parameters were analysed. Serum samples were immediately centrifuged and stored at −80°C until analysis. Based on the median values (93 mg/dL), patients were divided into low and high LDL‐C groups.

### 2.4 Clinical Follow‐Up

Patients were followed up until August 2018, when the database was censored. No patient in the subset analysed here (*n* = 241) was lost to follow‐up.

### 2.5 Statistical Analysis

Data are expressed as mean ± standard deviation or median (25th–75th percentile) for continuous variables and as frequencies and percentages for categorical variables. For continuous variables, unpaired two‐tailed Student's *t* test and Wilcoxon rank‐sum test were used to assess between two groups, as appropriate. A one‐way ANOVA and Kruskal–Wallis test were used to compare continuous variables between the four groups, as appropriate. Categorical comparisons were performed using Pearson's *χ* ^2^ test or Fisher's exact test as appropriate. Survival analysis was performed by applying the Kaplan–Meier method and the log‐rank test. The hazard ratio (HR) and 95% confidence interval (CI) were analysed with Cox proportional hazards regression models. Additionally, to explore the dose–response relationship between LDL‐C levels and all‐cause mortality, we categorized LDL‐C levels into sextiles and calculated HRs for each sextile using Cox proportional hazards models, with the highest LDL‐C sextile serving as the reference. Independent variables for model development were selected based on clinical relevance and the results of the univariate analysis in this study. In the low ASMI group, a backward stepwise regression was applied to refine the selection of independent variables and construct the final multivariate Cox regression model. To further validate the robustness of the findings, we conducted several subgroup and sensitivity analyses. First, we performed subgroup analysis based on HF phenotypes―HF with preserved ejection fraction (HFpEF: LVEF > 40%) and HF with reduced ejection fraction (HFrEF: LVEF ≤ 40%)—considering differences in clinical characteristics, including body composition and aetiology. Second, we performed additional subgroup analysis according to statin use. After comparing baseline characteristics of these subgroups, we performed survival analysis stratified by LDL‐C levels and ASMI within each subgroup. We further performed interaction tests to evaluate whether the associations of LDL‐C and ASMI with all‐cause mortality differed by HF phenotypes and statin use. Lastly, as a sensitivity analysis, we restricted our cohort to patients who survived at least 2 years from baseline and repeated the survival analyses to confirm whether the observed associations persisted thereafter. As missing data were minimal and deemed unlikely to affect the results substantially, no imputation was applied and all analyses were based on complete cases. Statistical analyses were performed with JMP Pro 16 (SAS Institute Inc., Cary, NC). A *p* value < 0.05 was considered statistically significant.

## 3 Results

### 3.1 Patient Characteristics

During a median follow‐up of 6.3 [4.0–7.3] years, 95 patients (39%) died. The baseline characteristics of the study population are shown in Table 1. The study cohort consisted of 192 males (80%) and 49 females. The mean age was 68 ± 11 years, and the mean LVEF was 39% ± 13%. Most patients were in New York Heart Association (NYHA) classes II–III, and the median NT‐proBNP level was 586 [224–1412] pg/mL. There were significant differences in age, sex, body mass index (BMI), LVEF, prevalence of hypertension, diabetes, CAD, atrial fibrillation, anaemia, muscle wasting, high‐density lipoprotein cholesterol (HDL‐C) levels, triglyceride (TG) levels, muscle strength, fat mass, peakVO_2_ and the use of β‐blockers, lipid‐lowering agents and aspirin between the four groups. Among the four groups, patients with low LDL‐C levels and low ASMI were the oldest; had the lowest BMI; the worst cardiac systolic function; the highest prevalence of CAD, atrial fibrillation and anaemia; the lowest TG levels and fat mass; and the lowest exercise capacity. Patients with low LDL‐C levels were significantly more likely to be male, have diabetes and CAD, worse kidney function, lower TG levels and higher prescription rates of β‐blockers, lipid‐lowering agents and aspirin use than those patients with high LDL‐C levels (Table S1).

**TABLE 1:** Baseline characteristics of patients by ASMI and LDL‐C levels.

Variables	Overall (*n* = 241)	Low ASMI (*n* = 121)		High ASMI (*n* = 120)		*p*
		Low LDL‐C (*n* = 62)	High LDL‐C (*n* = 59)	Low LDL‐C (*n* = 59)	High LDL‐C (*n* = 61)	
Age, years	68 ± 11	71 ± 9	70 ± 11	65 ± 12	65 ± 10	0.001
Male sex, *n* (%)	192 (79.7)	53 (85.5)	43 (72.9)	54 (91.5)	42 (68.9)	0.006
BMI, kg/m^2^	29.0 ± 5.1	26.2 ± 4.3	26.7 ± 4.5	32.0 ± 5.0	31.3 ± 4.0	< 0.0001
NYHA class	2.32 ± 0.63	2.42 ± 0.67	2.39 ± 0.59	2.32 ± 0.60	2.16 ± 0.64	0.13
LVEF, %	39 ± 13	35 ± 13	38 ± 12	40 ± 12	42 ± 15	0.04
HFpEF, *n* (%)	75 (31.1)	12 (19.3)	18 (30.5)	20 (33.9)	25 (41.0)	0.07
Comorbidities, *n* (%)						
Hypertension	193 (81.1)	52 (83.9)	38 (64.4)	50 (86.2)	53 (89.8)	0.002
Diabetes mellitus	92 (38.8)	23 (37.7)	15 (25.4)	32 (55.2)	22 (37.3)	0.01
Current smoking	29 (12.0)	6 (9.7)	8 (13.6)	8 (13.6)	7 (11.5)	0.90
CAD	140 (58.3)	51 (82.6)	30 (51.7)	34 (57.6)	24 (41.0)	< 0.0001
Atrial fibrillation	91 (37.8)	31 (50.0)	25 (42.4)	17 (28.8)	18 (29.5)	0.04
Anaemia	76 (31.5)	27 (43.6)	23 (39.0)	14 (23.7)	12 (19.7)	0.01
Muscle wasting	45 (18.7)	25 (40.3)	20 (33.9)	0 (0)	0 (0)	< 0.0001
Cardiac cachexia	56 (23.3)	14 (23.0)	10 (17.0)	14 (23.7)	18 (29.5)	0.45
Laboratory data						
High‐sensitivity CRP, mg/L	1.7 [1.0–3.4]	1.5 [0.8–3.5]	2.2 [1.3–3.4]	1.6 [0.8–3.3]	1.8 [1.1–3.7]	0.33
Creatinine, mg/dL	1.2 ± 0.4	1.3 ± 0.6	1.1 ± 0.3	1.2 ± 0.4	1.2 ± 0.4	0.08
Haemoglobin, g/dL	13.3 [12.4–14.5]	13.1 [12.0–14.2]	13.2 [12.1–14.2]	13.6 [13.0–14.7]	13.4 [12.8–14.8]	0.10
Albumin, g/L	37 [35–39]	37 [35–40]	36 [35–38]	37 [34–439]	37 [35–39]	0.66
NT‐proBNP, pg/mL	586 [224–1412]	860 [367–1588]	654 [258–2595]	431 [108–053]	516 [192–1317]	0.08
HDL‐C, mg/dL	45 [37–57]	48 [38–63]	48 [39–59]	41 [33–50]	47 [38–54]	0.01
LDL‐C, mg/dL	93 [70–122]	67 [58–77]	122 [106–159]	71 [59–84]	122 [107–136]	< 0.0001
TG, mg/dL	110 [83–169]	90 [71–117]	112 [88–154]	117 [88–190]	132 [95–187]	0.0006
Skeletal muscle and fat						
Handgrip strength, kg	38 ± 12	35 ± 10	33 ± 10	42 ± 11	40 ± 13	< 0.0001
Quadriceps strength, kg	39 ± 13	35 ± 11	35 ± 12	35 ± 13	43 ± 15	< 0.0001
ASMI, kg/m^2^	7.8 ± 1.1	7.0 ± 0.8	7.0 ± 0.6	8.8 ± 0.8	8.4 ± 0.9	< 0.0001
Fat mass, kg/m^2^	9.7 ± 3.6	8.3 ± 3.5	9.0 ± 3.8	10.4 ± 3.1	11.1 ± 3.5	< 0.0001
Functional capacity						
6‐min walk distance, m	422 ± 138	395 ± 128	406 ± 133	431 ± 159	454 ± 126	0.07
SPPB	11 [9–12]	11 [8–12]	11 [9–12]	11 [10–12]	12 [10–12]	0.32
PeakVO_2_, mL/min/kg	16.9 ± 5.0	14.8 ± 4.4	16.6 ± 4.6	17.8 ± 5.0	18.1 ± 5.3	0.002
Medication, *n* (%)						
ACE‐I or ARB	226 (93.8)	60 (96.8)	51 (86.4)	56 (94.9)	59 (96.7)	0.06
β‐Blockers	220 (91.3)	59 (95.2)	52 (86.4)	58 (98.3)	52 (85.3)	0.02
MRA	112 (46.5)	29 (46.8)	22 (37.3)	32 (54.2)	29 (47.5)	0.33
Loop diuretics	131 (54.6)	29 (46.8)	32 (55.2)	33 (55.9)	37 (60.7)	0.48
Statins	165 (68.5)	54 (87.1)	33 (56.9)	55 (93.2)	29 (47.5)	< 0.0001
Lipid‐lowering agents	171 (71.3)	54 (87.1)	33 (56.9)	55 (93.2)	62 (52.1)	< 0.0001
Aspirin	166 (68.9)	52 (83.9)	33 (55.9)	47 (79.7)	34 (55.7)	0.0002
Oral anticoagulants	82 (34.3)	24 (39.3)	21 (35.6)	17 (28.8)	20 (33.3)	0.67

*Note:* The values are numbers (percentages), means ± standard deviations or medians [25th–75th percentile range]. *p* values are derived from one‐way ANOVA or Kruskal–Wallis test for continuous variables and Pearson's *χ* ^2^ test or Fisher's exact test for categorical variables to assess differences among the four groups.

Abbreviations: ACE‐I, angiotensin‐converting enzyme inhibitor; ARB, angiotensin II receptor blocker; ASMI; appendicular skeletal muscle mass index; BMI, body mass index, aspartate transaminase; CAD, coronary artery disease; CRP, C‐reactive protein; HDL‐C, high‐density lipoprotein cholesterol; HFpEF, heart failure with preserved ejection fraction; LDL‐C, low‐density lipoprotein cholesterol; LVEF, left ventricular ejection fraction; MRA, mineral corticoid receptor antagonist; NT‐proBNP, N‐terminal pro‐B‐type natriuretic peptide; NYHA, New York Heart Association; SPPB, Short Physical Performance Battery; TG, triglyceride; VO_2_, oxygen consumption.

### 3.2 Impact of LDL‐C Levels and ASMI on Mortality

**FIGURE 2:**
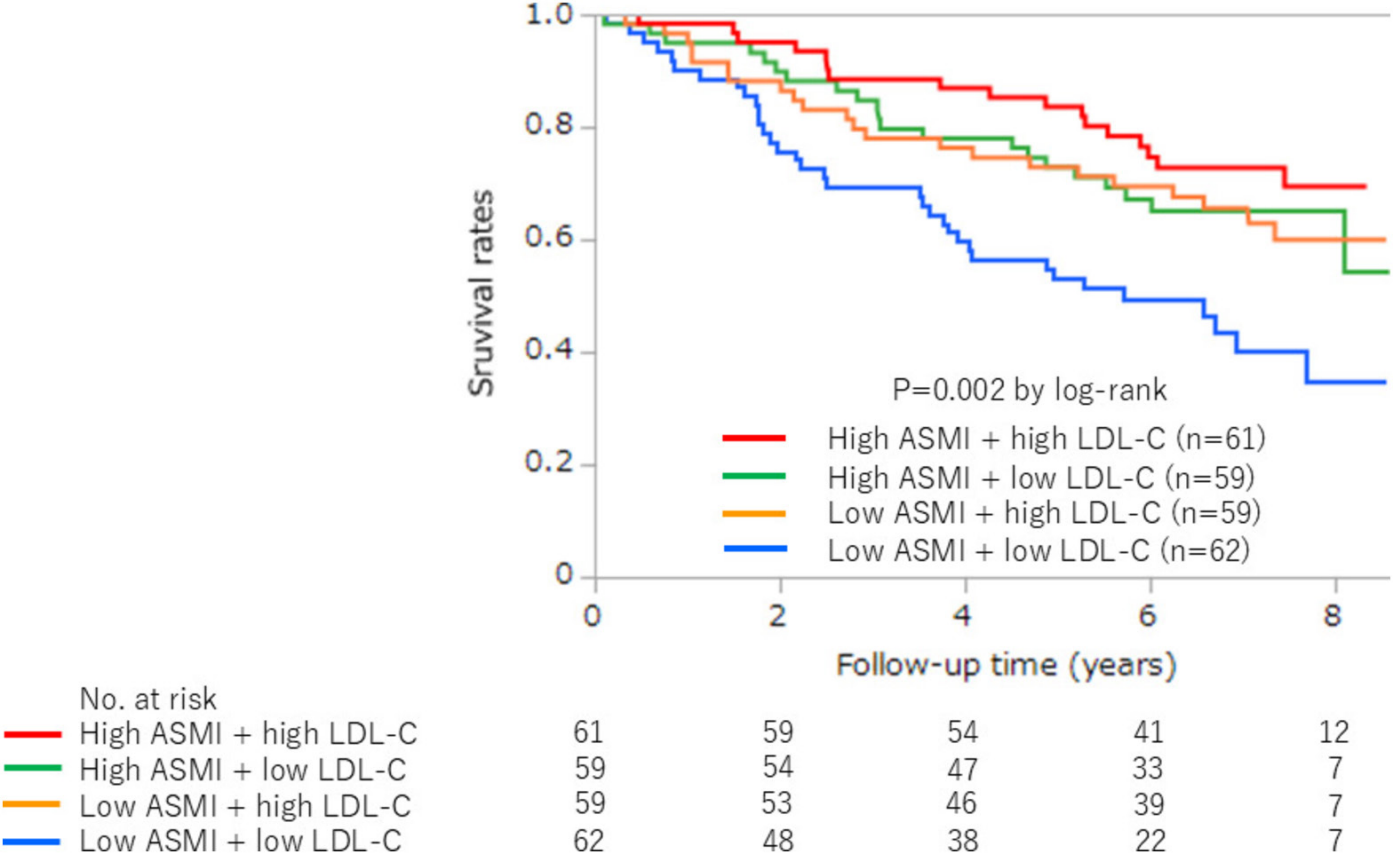
Kaplan–Meier survival curves according to ASMI and LDL‐C levels. ASMI, appendicular skeletal muscle mass index; LDL‐C, low‐density lipoprotein cholesterol.

**FIGURE 3:**
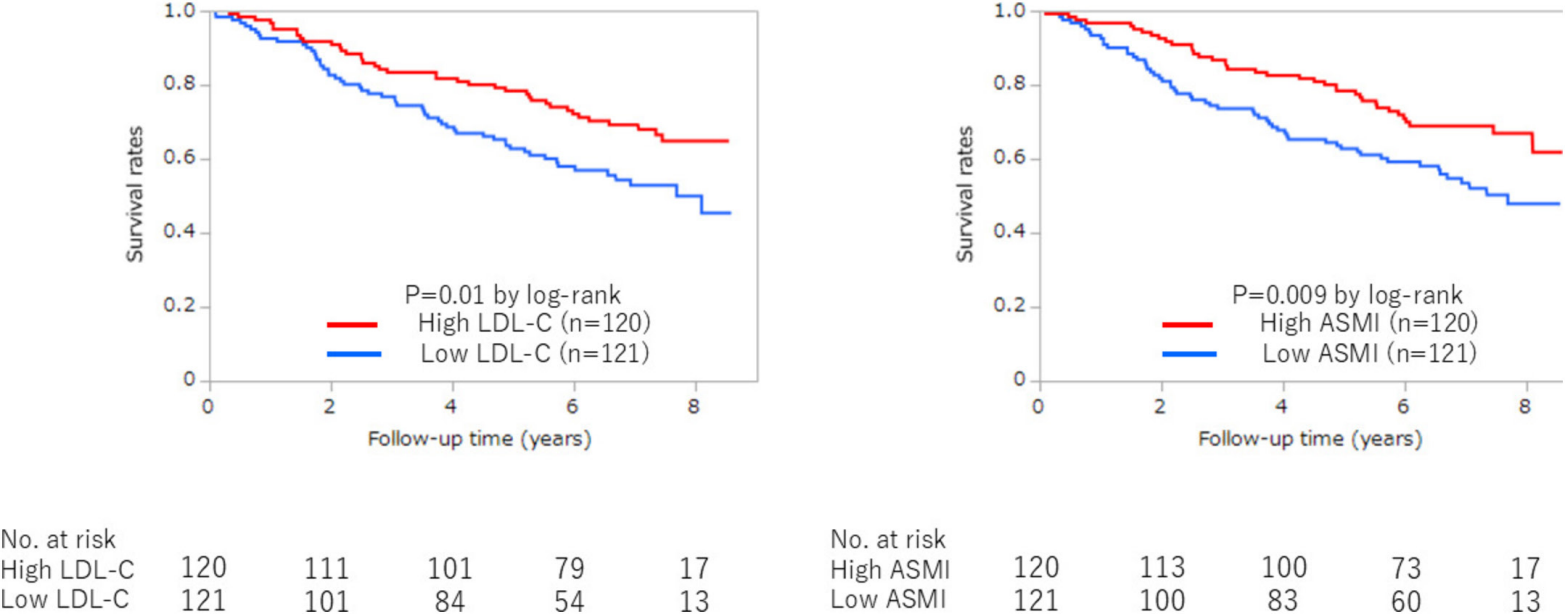
Impact of LDL‐C levels and ASMI on all‐cause mortality. ASMI, appendicular skeletal muscle mass index; LDL‐C, low‐density lipoprotein cholesterol.

**FIGURE 4:**
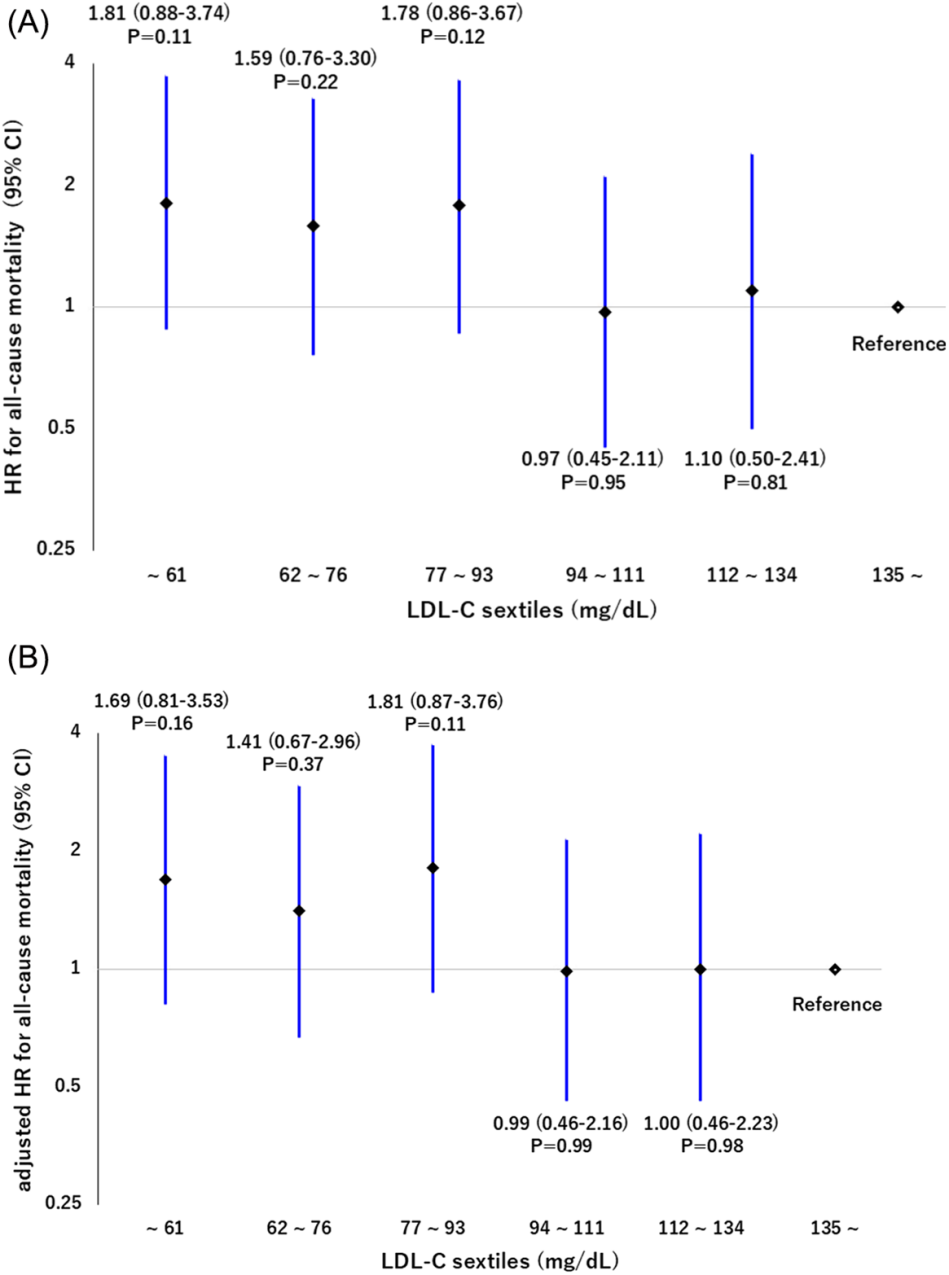
Dose–response relationship between LDL‐C levels and all‐cause mortality. Univariate analysis. Multivariate analysis adjusted for age, sex, BMI, creatinine and LVEF. BMI, body mass index; CI, confidence interval; HR hazard ration; LDL‐C, low‐density lipoprotein cholesterol; LVEF, left ventricular ejection fraction.

Kaplan–Meier analysis revealed significant differences in mortality between the four groups, with the highest mortality in patients with low LDL‐C levels with low ASMI and the lowest mortality in patients with high LDL‐C levels with high ASMI (*p* = 0.002, by log‐rank) (Figure 2). The mortality rate was significantly higher in patients with low LDL‐C levels compared with those with high LDL‐C levels (56 [46.3%] vs. 39 [32.5%], *p* = 0.01, by log‐rank) (Figure 3). Patients with low ASMI also had significantly higher mortality rates than those with high ASMI (57 [47.1%] vs. 38 [31.7%], *p* = 0.009, by log‐rank) (Figure 3). Multivariate Cox regression analysis confirmed that the low LDL‐C group remained significantly associated with higher mortality (HR 1.65, 95% CI 1.00–2.72, *p* = 0.04) (Table 2). In the dose–response relationship analysis between LDL‐C levels and all‐cause mortality, the univariate model showed numerically higher HRs (ranging from 1.59 to 1.81) in the lower LDL‐C groups (first to third sextiles) compared with the reference group, although not statistically significant. In contrast, the HRs of the higher LDL‐C groups (fourth and fifth sextiles) were nearly equivalent to the reference group (HRs ranging from 0.97 to 1.10) (Figure 4A). This trend was consistent in the multivariable model (Figure 4B).

**TABLE 2:** Univariate and multivariate Cox proportional hazard analysis for all‐cause mortality.

	All‐cause mortality		
	HR	95% CI	*p*
Univariate model (low LDL‐C group)	1.69	1.12–2.54	0.01
Multivariate Model 1	1.62	1.07–2.47	0.02
Adjusted for age, sex and BMI			
Multivariate Model 2	1.55	1.02–2.37	0.04
Adjusted for Model 1			
+Creatinine and anaemia			
Multivariate Model 3	1.65	1.00–2.72	0.04
Adjusted for Model 2			
+LVEF, NYHA class, CAD and lipid‐lowering agents			

Abbreviations: BMI, body mass index; CAD, coronary artery disease; CI, confidence interval; HR, hazard ratio; LDL‐C, low‐density lipoprotein cholesterol; LVEF, left ventricular ejection fraction; NYHA, New York Heart Association.

### 3.3 Impact of Low LDL‐C Levels on Mortality According to the ASMI Value

**FIGURE 5:**
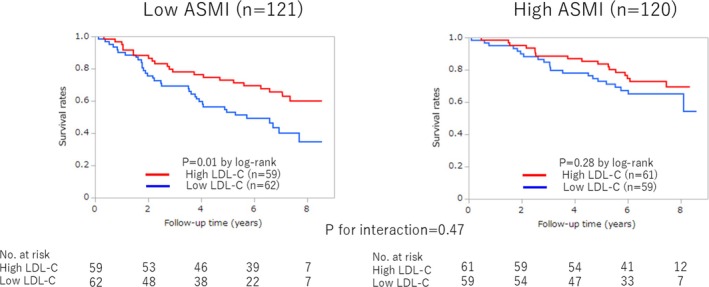
Kaplan–Meier survival curves according to ASMI and LDL‐C levels. ASMI, appendicular skeletal muscle mass index; LDL‐C, low‐density lipoprotein cholesterol.

The prognostic impact of low LDL‐C levels differed between the low and high ASMI groups, with statistical significance observed in the low ASMI group but not in the high ASMI group (56% vs. 37%, *p* = 0.01, and 36% vs. 28%, *p* = 0.28, by log‐rank: *p* for interaction = 0.47) (Figure 5).

### 3.4 Impact of LDL‐C Levels on Mortality in the Low ASMI Group

On univariate analysis in the low ASMI group, several factors were associated with higher mortality: older age, male sex, higher NYHA class, lower LVEF, diabetes, CAD, atrial fibrillation, higher creatinine and NT‐proBNP levels, lower haemoglobin and LDL‐C levels, lower muscle strength, lower fat mass, lower exercise capacity and higher rates of loop diuretic and aspirin use (Table S2). Several multivariate Cox regression hazards models confirmed that lower LDL‐C levels were significant predictors of mortality (Model 1 adjusted for age, sex, NYHA class, creatinine and haemoglobin; Model 2 adjusted for NYHA class, LVEF, CAD, quadriceps strength and fat mass; Model 3 adjusted for age, LVEF, creatinine, haemoglobin and fat mass; aHR 1.46–1.48 per 1 SD decrease in LDL‐C, all *p* < 0.05) (Table 3).

**TABLE 3:** Univariate and multivariate Cox proportional hazard analysis for all‐cause mortality in the low ASMI group.

	All‐cause mortality		
	HR	95% CI	*p*
Univariate model (per 1 SD decrease in LDL‐C)	1.65	1.21–2.31	0.003
Multivariate Model 1	1.46	1.04–2.12	0.04
Adjusted for age, sex, NYHA class, creatinine and haemoglobin			
Multivariate Model 2	1.48	1.00–2.24	0.04
Adjusted for NYHA class, LVEF, CAD, quadriceps strength and fat mass			
Multivariate Model 3	1.48	1.05–2.16	0.03
Adjusted for age, LVEF, creatinine, haemoglobin and fat mass			

Abbreviations: CAD, coronary artery disease; CI, confidence interval; HR, hazard ratio; LDL‐C, low‐density lipoprotein cholesterol; LVEF, left ventricular ejection fraction; NYHA, New York Heart Association; SD, standard deviation.

### 3.5 Comparison of the Factors Associated With Mortality Between the Four Groups

Overall, NYHA class, LVEF, kidney function, muscle strength and exercise capacity were associated with all‐cause mortality across the four groups (Table 4). While CAD tended to be associated with higher all‐cause mortality in the two groups with high LDL‐C levels, this association was not observed in the two groups with low LDL‐C levels. Older age, lower haemoglobin levels and lower fat mass were significantly associated with higher mortality only in the group with low LDL‐C levels and low ASMI.

**TABLE 4:** Differences in factors associated with all‐cause mortality by ASMI and LDL‐C levels.

Variables	Low ASMI and low LDL‐C (*n* = 62)			Low ASMI and high LDL‐C (*n* = 59)			High ASMI and low LDL‐C (*n* = 59)			High ASMI and high LDL‐C (*n* = 61)		
	HR	95% CI	*p*	HR	95% CI	*p*	HR	95% CI	*p*	HR	95% CI	*p*
Age, per 1 year	1.07	1.02–1.12	0.008	1.00	0.97–1.04	0.81	1.02	0.98–1.07	0.33	1.02	0.97–1.07	0.49
Male sex	2.04	0.62–6.69	0.24	2.03	0.69–6.01	0.20	—	—	0.99	0.82	0.30–2.21	0.69
BMI, per 1 kg/m^2^	0.94	0.87–1.01	0.09	1.01	0.91–1.10	0.85	1.01	0.92–1.10	0.85	1.04	0.93–1.17	0.48
NYHA class, per 1 class increase	1.38	0.82–2.41	0.23	3.46	1.67–7.32	0.001	2.93	1.33–6.95	0.01	1.74	0.81–3.95	0.16
LVEF, per 10% increase	0.73	0.52–0.98	0.04	0.58	0.36–0.87	0.01	0.53	0.36–0.78	0.002	0.71	0.49–0.99	0.04
Hypertension (present)	2.66	0.81–8.77	0.11	1.00	0.42–2.40	0.99	0.49	0.18–1.34	0.17	0.77	0.18–3.38	0.73
Diabetes mellitus (present)	1.56	0.79–3.10	0.20	1.76	0.73–4.20	0.20	0.58	0.24–1.39	0.22	1.64	0.63–4.26	0.31
Current smoking (present)	0.55	0.13–2.30	0.41	4.35	1.76–10.76	0.002	1.33	0.44–4.08	0.61	1.19	0.27–5.21	0.82
CAD (present)	0.97	0.40–2.36	0.95	3.07	1.19–7.94	0.02	1.53	0.62–3.80	0.36	2.33	0.89–6.15	0.09
Atrial fibrillation (present)	1.44	0.74–2.80	0.29	2.23	0.95–5.20	0.06	1.78	0.73–4.29	0.20	3.09	1.19–8.04	0.02
Anaemia (present)	2.18	1.12–4.25	0.02	1.06	0.45–2.49	0.89	3.71	1.52–9.02	0.004	0.93	0.27–3.24	0.91
Muscle wasting (present)	0.78	0.39–1.56	0.48	3.28	1.41–7.61	0.006	—	—	—	—	—	—
Cardiac cachexia (present)	1.05	0.48–2.31	0.90	1.26	0.43–3.75	0.67	2.28	0.92–5.60	0.07	1.53	0.56–4.15	0.40
Hs‐CRP, per 1‐mg/L increase	1.07	0.83–1.36	0.57	1.26	0.99–1.57	0.053	0.93	0.65–1.23	0.63	1.01	0.76–1.28	0.97
Creatinine, per 0.1‐mg/dL increase	1.03	0.97–1.08	0.25	1.18	1.06–1.31	0.003	1.24	1.11–1.38	< 0.0001	1.08	0.98–1.17	0.09
Haemoglobin, per 1‐g/dL increase	0.77	0.60–0.98	0.04	0.81	0.57–1.13	0.22	0.86	0.67–1.14	0.27	0.92	0.68–1.26	0.62
Albumin, per 1‐g/L increase	0.93	0.85–1.03	0.15	0.96	0.89–1.06	0.36	0.97	0.87–1.08	0.57	1.04	0.91–1.20	0.61
NT‐proBNP, per 1 SD increase	3.46	1.81–6.33	< 0.0001	1.50	1.12–1.91	0.002	2.03	1.34–3.72	0.001	1.13	0.54–1.63	0.62
HDL‐C, per 1 SD increase	0.89	0.60–1.22	0.53	0.52	0.25–0.96	0.06	0.62	0.31–1.18	0.16	0.62	0.31–1.18	0.16
LDL‐C, per 1 SD increase	0.91	0.31–2.93	0.87	0.46	0.20–0.87	0.04	1.29	0.44–4.09	0.65	1.61	0.89–2.59	0.07
TG, per 1 SD increase	0.95	0.57–1.44	0.82	0.87	0.51–1.23	0.53	0.55	0.27–0.97	0.04	1.82	1.19–2.65	0.003
Handgrip strength, per 1‐kg increase	0.96	0.91–0.99	0.04	1.02	0.98–1.06	0.25	0.99	0.95–1.03	0.61	0.95	0.91–0.99	0.03
Quadriceps strength, per 1‐kg increase	0.98	0.95–1.01	0.28	0.95	0.91–0.99	0.04	0.98	0.94–1.02	0.35	0.95	0.92–0.99	0.01
ASMI, per 1‐kg/m^2^ increase	1.07	0.70–1.72	0.78	0.93	0.49–1.79	0.82	1.48	0.87–2.42	0.13	0.85	0.50–1.43	0.55
Fat mass, per 1‐kg/m^2^ increase	0.90	0.81–0.99	0.03	0.97	0.85–1.09	0.61	0.97	0.84–1.12	0.69	1.04	0.91–1.19	0.55
6‐min walk distance, per 10‐m increase	0.97	0.95–1.00	0.053	0.95	0.92–0.98	0.0004	0.98	0.95–1.01	0.20	0.97	0.93–1.00	0.06
SPPB, per 1‐point increase	0.84	0.73–0.97	0.01	0.82	0.73–0.94	0.002	0.59	0.44–0.79	0.0003	0.92	0.74–1.21	0.48
PeakVO_2_, per 1‐mL/min/kg increase	0.97	0.89–1.05	0.44	0.87	0.81–0.94	< 0.0001	0.93	0.83–1.03	0.15	0.90	0.81–0.99	0.03
ACE‐I/ARB (present)	0.11	0.02–0.48	0.004	1.05	0.31–3.54	0.94	—	—	0.99	—	—	0.99
β‐Blockers (present)	0.58	0.14–2.42	0.45	1.71	0.40–7.34	0.47	—	—	0.99	1.36	0.31–5.96	0.68
MRA (present)	1.07	0.55–2.10	0.83	2.14	0.92–4.98	0.08	2.47	0.96–6.38	0.06	1.34	0.52–3.47	0.55
Loop diuretics (present)	1.47	0.76–2.87	0.25	3.58	1.32–9.73	0.01	1.14	0.48–2.71	0.77	3.68	1.05–12.86	0.04
Statins (present)	0.54	0.22–1.32	0.18	1.09	0.47–2.51	0.85	0.58	0.15–2.85	0.58	1.91	0.73–5.04	0.19
Lipid‐lowering agents (present)	0.54	0.22–1.32	0.18	0.78	0.33–1.84	0.57	0.58	0.15–2.85	0.58	2.37	0.87–6.43	0.09
Aspirin (present)	1.70	0.60–4.83	0.32	1.70	0.71–4.07	0.23	1.05	0.35–3.11	0.93	0.66	0.26–1.72	0.40
Oral anticoagulants (present)	0.99	0.50–1.97	0.98	1.77	0.76–4.09	0.18	0.98	0.38–2.52	0.96	4.34	1.60–11.77	0.004

Abbreviations: ACE‐I, angiotensin‐converting enzyme inhibitor; ARB, angiotensin II receptor blocker; ASMI; appendicular skeletal muscle mass index; BMI, body mass index, aspartate transaminase; CAD, coronary artery disease; CRP, C‐reactive protein; HDL‐C, high‐density lipoprotein cholesterol; HR, hazard ratio; LDL‐C, low‐density lipoprotein cholesterol; LVEF, left ventricular ejection fraction; MRA, mineral corticoid receptor antagonist; NT‐proBNP, N‐terminal pro‐B‐type natriuretic peptide; NYHA, New York Heart Association; SD, standard deviation; SPPB, Short Physical Performance Battery; TG, triglycerides; VO_2_, oxygen consumption.

### 3.6 Subgroup and Sensitivity Analyses

In the subgroup analysis based on HF phenotype, patients with HFpEF were significantly older, had a lower proportion of males, a higher BMI, higher LDL‐C levels and a lower prevalence of muscle wasting and cardiac cachexia compared with those with HFrEF (Table S3). When stratified by LDL‐C level and ASMI, a significant difference in all‐cause mortality was observed in the HFrEF subgroup (*p* = 0.03, by log‐rank) but not in the HFpEF subgroup (*p* = 0.56, by log‐rank). Patients with low ASMI and low LDL‐C levels demonstrated the worst survival (Figure S1A,B). Although low LDL‐C levels and low ASMI were each significantly associated with higher all‐cause mortality only in the HFrEF subgroup (low LDL‐C: *p* = 0.04, by log‐rank; low ASMI: p = 0.04, by log‐rank), interaction analyses revealed no statistically significant differences in these associations between HF phenotypes (interaction *p* = 0.94 for LDL‐C, interaction *p* = 0.69 for ASMI; Figure S2A,B). In the HFrEF subgroup, multivariate Cox proportional hazards analysis adjusted for age and sex revealed a significant association between low LDL‐C levels and higher all‐cause mortality (aHR 1.59, 95% CI 1.00–2.52, *p* = 0.04). When further stratified by ASMI, this association showed a trend towards significance in the low ASMI group (*p* = 0.08, by log‐rank) but not in the high ASMI group (*p* = 0.32, by log‐rank) (Figure S3).

In the subgroup analysis according to statin use, patients taking statins had a significantly higher proportion of males, a higher BMI, a higher ASMI, lower LDL‐C levels and a higher prevalence of CAD than those not taking statins (Table S4). When stratified by LDL‐C level and ASMI, a significant difference in all‐cause mortality was observed in the nonstatin subgroup (*p* = 0.003, by log‐rank) but not in the statin subgroup (*p* = 0.09, by log‐rank). Patients with low ASMI and low LDL‐C levels demonstrated the worst survival (Figure S4A,B). Specifically, in the nonstatin subgroup, low LDL‐C levels and low ASMI were each significantly associated with higher all‐cause mortality (low LDL‐C: *p* = 0.002, by log‐rank; low ASMI: *p* = 0.04, by log‐rank). Interaction analyses revealed a borderline significant difference in the association between low LDL‐C and mortality according to statin use (interaction *p* = 0.06; Figure S5A,B). In contrast, no significant interaction was observed in the association between low ASMI and mortality according to statin use (interaction *p* = 0.47; Figure S5A,B). In the nonstatin subgroup, multivariate Cox proportional hazards analysis adjusted for age and sex revealed a significant association between low LDL‐C levels and higher all‐cause mortality (aHR 3.45, 95% CI 1.48–8.02, *p* = 0.004). When further stratified by ASMI, this association was significant in the low ASMI group (*p* = 0.02, by log‐rank) but not in the high ASMI group (*p* = 0.14, by log‐rank) (Figure S6).

In a sensitivity analysis restricted to patients who survived beyond 2 years, no statistically significant differences in all‐cause mortality were observed among the four groups stratified by LDL‐C levels and ASMI (*p* = 0.11, by log‐rank), although patients with low ASMI with low LDL‐C exhibited numerically the worst survival (Figure S7). The low LDL‐C group showed a trend towards higher all‐cause mortality compared with the high LDL‐C group (*p* = 0.10, by log‐rank) (Figure S8), particularly in the low ASMI group (*p* = 0.07, by log‐rank) but not in the high ASMI group (*p* = 0.53, by log‐rank; Figure S9).

## 4 Discussion

Our study is the first to demonstrate an association between LDL‐C levels and mortality in patients with chronic HF in the context of skeletal muscle mass. It has three main findings. First, low LDL‐C levels are significantly associated with higher all‐cause mortality in patients with chronic HF. Second, this paradoxical relationship is predominantly observed in patients with low ASMI. Third, lower haemoglobin levels and lower fat mass are significantly associated with higher mortality only in patients with low LDL‐C levels and low ASMI.

In the present study, patients with low LDL‐C levels had significantly higher mortality even after adjustment for potential confounders. In addition, a nonlinear relationship or threshold effect between LDL‐C levels and mortality was suggested. Elevated LDL‐C levels drive atherosclerosis, and intensive LDL‐C lowering therapies, including statins and PCSK‐9 inhibitors, are highly effective [2, 3, 4]. At the same time, cholesterol is indispensable for normal physiology, serving as a structural component of cell membranes and as a precursor for steroid hormones, bile acids, vitamin D and other essential molecules [15, 29]. Plasma lipoproteins also play a protective role by neutralizing bacterial lipopolysaccharides and modulating immune responses in sepsis [16, 30]. In skeletal muscle, the cholesterol‐rich transverse tubule (T‐tubule) system contributes to excitation–contraction coupling and glucose metabolism [15]. Cholesterol depletion has been linked to impaired contractility and structural changes in muscle fibres, providing a potential mechanism for statin‐induced myopathy [31, 32, 33, 34]. These findings emphasize that LDL‐C is not only a driver of atherosclerosis but also crucial for maintaining physiological integrity, which may help explain the cholesterol survival paradox observed in various pathological conditions, including HF [7, 8, 9, 35, 36].

The association between low LDL‐C levels and higher mortality was particularly evident in the low ASMI group, while low LDL‐C levels had no significant prognostic impact in the high ASMI group. We previously demonstrated that patients with low LDL‐C levels at the onset of acute coronary syndrome had significantly worse prognosis than those with high LDL‐C levels [7]. Furthermore, physical frailty was a poor prognostic factor only in patients with low LDL‐C levels [7]. The present findings extend this knowledge in the context of HF, re‐emphasizing the importance of body composition assessment and further suggesting that the combined assessment of LDL‐C levels and skeletal muscle mass may help stratify the risk in patients with chronic HF.

The factors associated with mortality in patients with low LDL‐C levels and low ASMI were distinct when compared with other groups, with lower haemoglobin levels and lower fat mass being significantly associated with higher mortality. Cholesterol is a primary component of the platelet membrane lipid rafts, and its depletion induces raft disruption, impairing platelet aggregation [37]. In addition, LDL‐C depletion leads to lower serum PCSK‐9 concentrations [38], and PCSK‐9, beyond regulating LDL‐C receptors, also promotes platelet activation via the PCSK‐9/CD36 pathway [39]. These mechanisms may underlie the link between low LDL‐C and bleeding risk [40, 41], with muscle wasting potentially exaggerating this effect. This potential link is further supported by previous findings that demonstrate an association between muscle wasting and an increased bleeding risk [42]. While the prognostic impact of obesity, as defined by a high BMI, remains contentious, excess adiposity is generally associated with worse outcomes in HF [43, 44, 45]. Conversely, lower fat mass has been shown to be associated with higher mortality in patients with HF [18], presumably due to its protective effects, such as energy reserves and adipocytokine secretion [46, 47]. In this study, patients with low LDL‐C levels and low ASMI had the lowest fat mass, which was significantly associated with higher mortality. In contrast, higher fat mass showed a trend towards increased risk in those with high LDL‐C levels and high ASMI. These findings raise a hypothesis that the prognostic impact of adipose tissue in HF might exhibit a U‐shaped curve depending on its quantity; however, further research with adequate statistical power is needed to confirm this hypothesis.

Subgroup analysis by HF phenotype and sensitivity analysis generally supported the association between low LDL‐C levels and higher all‐cause mortality in the context of skeletal muscle, with this relationship being particularly evident in the HFrEF group. Significant differences in BMI, body fat and the prevalence of muscle wasting and cardiac cachexia were observed between HFrEF and HFpEF, suggesting that differences in metabolic reserve across HF phenotypes may modulate the extent of the adverse effects of low cholesterol levels and reduced skeletal muscle mass. Although no statistically significant interactions were observed, given the heterogeneity of HFpEF and its frequent comorbidities, the prognostic significance of LDL‐C warrants further investigation according to HF phenotypes. In subgroup analysis by statin use, this association tended to be more pronounced only in the nonstatin subgroup. Compared with the nonstatin subgroup, the statin subgroup exhibited significantly higher CAD prevalence and significantly lower inflammatory responses. Given these findings, statins might mitigate the potential adverse effects associated with low LDL‐C levels through their antiatherosclerotic and pleiotropic effects, particularly in ischemic HF [48]. However, whether the adverse impact of low LDL‐C levels depends on HF aetiology or pathophysiology remains unclear, and further investigation is warranted.

This study has several limitations. First, the present study involved a small sample of patients with chronic stable HF. Although the follow‐up period was relatively long, the number of clinical events was limited, reducing the statistical power and resulting in wide CIs. This statistical imprecision made it challenging to perform robust multivariate analyses, particularly in subgroup and sensitivity analyses. Therefore, these findings should be interpreted cautiously. To address the clinical impact of LDL‐C levels in the context of skeletal muscle mass, further large‐scale studies, including patients with more advanced HF, are warranted. Second, there is a lack of data on cancer. Dysregulation of the cholesterol synthesis pathway and its metabolites is strongly involved in the development, proliferation and self‐renewal of various cancer tissues [49]. At the same time, a significant association between low serum cholesterol levels and worse cancer survival has also been shown [50]. Furthermore, cancer causes muscle wasting through chronic inflammation, altered energy balance and anorexia [51, 52]. Detailed data on cancer should be incorporated to better understand the potential clinical impact of the present findings. Third, serum creatinine was used to evaluate kidney function. However, serum creatinine is produced by skeletal muscle and thus its levels are influenced by muscle mass, with lower values observed in conditions of muscle wasting [53]. This may have inevitably affected the results of our multivariable analyses. Therefore, it would be desirable to reassess our findings using alternative biomarkers of kidney function, such as cystatin C, which are less influenced by skeletal muscle mass [54]. Lastly, although total body fat mass was measured by DEXA, the distribution of adipose tissue (i.e., subcutaneous and visceral fat) could not be assessed. Compared with subcutaneous fat, visceral fat is a better predictor of cardiometabolic risks such as inflammation and insulin resistance [55]. Some studies of patients with cardiovascular disease have even shown that the ratio of visceral fat to subcutaneous fat is more strongly associated with poor clinical outcomes than visceral fat alone [56, 57]. Considering abnormal fat distribution may enable a more in‐depth investigation of the protective effects of adipose tissue in HF patients with low LDL‐C levels and low ASMI.

## 5 Conclusions

In patients with chronic stable HF, low LDL‐C levels are associated with increased mortality. Although no statistically significant interaction with skeletal muscle mass was observed, this association appeared to be more pronounced in patients with reduced skeletal muscle mass. A plausible explanation is that cholesterol may exert protective effects by maintaining cell membrane integrity, serving as a precursor for essential biological molecules, modulating immune responses and playing a pivotal role in maintaining skeletal muscle mass and function. Additionally, the adverse impact of low LDL‐C levels on mortality was primarily observed in patients not receiving statins, suggesting that statins may mitigate this risk through both antiatherosclerotic and pleiotropic effects, particularly in ischemic HF. Overall, the combined assessment of LDL‐C levels and skeletal muscle mass may be useful for risk stratification in chronic HF patients. Further research is required to elucidate the mechanisms underlying the cholesterol paradox and to determine the optimal LDL‐C levels for patients with chronic HF.

## Funding

The project was supported by the European Commission's 7th Framework Programme (FP7/2003–2013) under grant agreement number 241558 and the Russian Ministry of Science and Education within the FTP ‘R&D in priority fields of the S&T complex of Russia 2007–2012’ under state contract number 02.527.11.0007.

## Conflicts of Interest

R.S. reports grants from Japan Heart Foundation/Bayer Yakuhin Research Grant Abroad, during the conduct of the study. S.D.A. reports grants and personal fees from Vifor and Abbott Vascular and personal fees for consultancies, trial committee work and/or lectures from Actimed, Amgen, Astra Zeneca, Bayer, Boehringer Ingelheim, Bioventrix, Brahms, Cardiac Dimensions, Cardior, Cordio, CVRx, Edwards, Farraday, Impulse Dynamics, Janssen, Novartis, Occlutech, Pfizer, Respicardia, Servier, Vectorious and V‐Wave and declares that he is named co‐inventor of two patent applications regarding MR‐proANP (DE 102007010834 and DE 102007022367), but he does not benefit personally from the related issued patents. S.v.H. has been a paid consultant for and/or received honoraria payments from AstraZeneca, Bayer, Boehringer Ingelheim, BRAHMS, Edwards Lifesciences, Lumira DX, Novartis, Novo Nordisk, Pharmacosmos, Respicardia and CSL Vifor. S.v.H. reports research support from Amgen, AstraZeneca, Boehringer Ingelheim, Pharmacosmos, IMI and the German Center for Cardiovascular Research (DZHK).

## Supporting information


**Table S1:** Baseline characteristics of patients by low and high LDL‐C levels.
**Table S2:** Factors associated with all‐cause mortality in the low ASMI group.
**Table S3:** Baseline characteristics of patients stratified by HF phenotype.
**Table S4:** Baseline characteristics of patients stratified by statin use.

**Figure S1:** Kaplan–Meier survival curves according to ASMI and LDL‐C levels. (A) HFpEF subgroup. (B) HFrEF subgroup. ASMI, appendicular skeletal muscle mass index; HFpEF, heart failure with preserved ejection fraction; HFrEF, heart failure with reduced ejection fraction; LDL‐C, low‐density lipoprotein cholesterol.
**Figure S2:** Impact of LDL‐C levels (A) and ASMI (B) on all‐cause mortality in patients with HFpEF and HFrEF. ASMI, appendicular skeletal muscle mass index; HFpEF, heart failure with preserved ejection fraction; HFrEF, heart failure with reduced ejection fraction; LDL‐C, low‐density lipoprotein cholesterol.
**Figure S3:** Impact of LDL‐C levels on all‐cause mortality according to the ASMI value in patients with HFrEF. ASMI, appendicular skeletal muscle mass index; HFrEF, heart failure with reduced ejection fraction; LDL‐C, low‐density lipoprotein cholesterol.
**Figure S4:** Kaplan–Meier survival curves according to ASMI and LDL‐C levels. (A) Statin subgroup. (B) Nonstatin subgroup. ASMI, appendicular skeletal muscle mass index; LDL‐C, low‐density lipoprotein cholesterol.
**Figure S5:** Impact of LDL‐C levels (A) and ASMI (B) on all‐cause mortality in patients with or without statin use. ASMI, appendicular skeletal muscle mass index; LDL‐C, low‐density lipoprotein cholesterol.
**Figure S6:** Impact of LDL‐C levels on all‐cause mortality according to the ASMI value in the nonstatin subgroup. ASMI, appendicular skeletal muscle mass index; LDL‐C, low‐density lipoprotein cholesterol.
**Figure S7:** Kaplan–Meier survival curves according to ASMI and LDL‐C levels (patients surviving > 2 years). ASMI, appendicular skeletal muscle mass index; LDL‐C, low‐density lipoprotein cholesterol.
**Figure S8:** Impact of LDL‐C levels and ASMI on all‐cause mortality (patients surviving > 2 years). ASMI, appendicular skeletal muscle mass index; LDL‐C, low‐density lipoprotein cholesterol.
**Figure S9:** Impact of LDL‐C levels on all‐cause mortality according to the ASMI value (patients surviving > 2 years). ASMI, appendicular skeletal muscle mass index; LDL‐C, low‐density lipoprotein cholesterol.

**Data S1:** Supporting Information.

## References

[1] World Health Organization , “Indicator Metadata Registry List: Raised Cholesterol,” (2025), https://www.who.int/data/gho/indicator‐metadata‐registry/imr‐details/3236.

[2] S. S. Virani , L. K. Newby , S. V. Arnold , et al., “2023 AHA/ACC/ACCP/ASPC/NLA/PCNA Guideline for the Management of Patients With Chronic Coronary Disease: A Report of the American Heart Association/American College of Cardiology Joint Committee on Clinical Practice Guidelines,” Circulation 148 (2023): e9–e119. 37471501 10.1161/CIR.0000000000001168

[3] R. A. Byrne , X. Rossello , J. J. Coughlan , E. Barbato , C. Berry , and A. Chieffo , “2023 ESC Guidelines for the Management of Acute Coronary Syndromes,” European Heart Journal 44 (2023): 3720–3826. 37622654 10.1093/eurheartj/ehad191

[4] C. Vrints , F. Andreotti , K. C. Koskinas , et al., “2024 ESC Guidelines for the Management of Chronic Coronary Syndromes,” European Heart Journal 45 (2024): 3415–3537. 39210710 10.1093/eurheartj/ehae177

[5] Y. C. Klimentidis , A. Arora , M. Newell , et al., “Phenotypic and Genetic Characterization of Lower LDL Cholesterol and Increased Type 2 Diabetes Risk in the UK Biobank,” Diabetes 69 (2020): 2194–2205. 32493714 10.2337/db19-1134 PMC7506834

[6] S. L. Harrison , D. A. Lane , M. Banach , et al., “Lipid Levels, Atrial Fibrillation and the Impact of Age: Results From the LIPIDOGRAM2015 Study,” Atherosclerosis 312 (2020): 16–22. 32947222 10.1016/j.atherosclerosis.2020.08.026

[7] R. Sato , Y. Matsuzawa , T. Yoshii , et al., “Impact of Low‐Density Lipoprotein Cholesterol Levels at Acute Coronary Syndrome Admission on Long‐Term Clinical Outcomes,” Journal of Atherosclerosis and Thrombosis 31 (2024): 444–460. 37821363 10.5551/jat.64368 PMC10999725

[8] M. Rauchhaus , A. L. Clark , W. Doehner , et al., “The Relationship Between Cholesterol and Survival in Patients With Chronic Heart Failure,” Journal of the American College of Cardiology 42 (2003): 1933–1940. 14662255 10.1016/j.jacc.2003.07.016

[9] R. Gouveia , S. Madureira , C. Elias , et al., “Lower Low Density Lipoprotein Cholesterol Associates to Higher Mortality in Non‐Diabetic Heart Failure Patients,” International Journal of Cardiology Cardiovascular Risk and Prevention 18 (2023): 200197. 37521244 10.1016/j.ijcrp.2023.200197 PMC10374454

[10] J. Kjekshus , E. Apetrei , V. Barrios , et al., “Rosuvastatin in Older Patients With Systolic Heart Failure,” New England Journal of Medicine 357 (2007): 2248–2261. 17984166 10.1056/NEJMoa0706201

[11] L. Tavazzi , A. P. Maggioni , R. Marchioli , et al., “Effect of Rosuvastatin in Patients With Chronic Heart Failure (The GISSI‐HF Trial): A Randomised, Double‐Blind, Placebo‐Controlled Trial,” Lancet (London, England) 372 (2008): 1231–1239. 18757089 10.1016/S0140-6736(08)61240-4

[12] T. A. McDonagh , M. Metra , M. Adamo , et al., “2021 ESC Guidelines for the Diagnosis and Treatment of Acute and Chronic Heart Failure,” European Heart Journal 42 (2021): 3599–3726. 34447992 10.1093/eurheartj/ehab368

[13] P. A. Heidenreich , B. Bozkurt , D. Aguilar , et al., “2022 AHA/ACC/HFSA Guideline for the Management of Heart Failure: A Report of the American College of Cardiology/American Heart Association Joint Committee on Clinical Practice Guidelines,” Circulation 145 (2022): e895–e1032. 35363499 10.1161/CIR.0000000000001063

[14] G. Barrientos , P. Llanos , J. Hidalgo , et al., “Cholesterol Removal From Adult Skeletal Muscle Impairs Excitation‐Contraction Coupling and Aging Reduces Caveolin‐3 and Alters the Expression of Other Triadic Proteins,” Frontiers in Physiology 6 (2015): 105. 25914646 10.3389/fphys.2015.00105 PMC4392612

[15] G. Barrientos , P. Sánchez‐Aguilera , E. Jaimovich , C. Hidalgo , and P. Llanos , “Membrane Cholesterol in Skeletal Muscle: A Novel Player in Excitation‐Contraction Coupling and Insulin Resistance,” Journal of Diabetes Research 2017 (2017): 3941898. 28367451 10.1155/2017/3941898 PMC5358446

[16] S. von Haehling , J. C. Schefold , J. Springer , and S. D. Anker , “The Cholesterol Paradox Revisited: Heart Failure, Systemic Inflammation, and Beyond,” Heart Failure Clinics 4 (2008): 141–151. 18433694 10.1016/j.hfc.2008.01.009

[17] S. von Haehling , T. Garfias Macedo , M. Valentova , et al., “Muscle Wasting as an Independent Predictor of Survival in Patients With Chronic Heart Failure,” Journal of Cachexia, Sarcopenia and Muscle 11 (2020): 1242–1249. 32767518 10.1002/jcsm.12603 PMC7567155

[18] M. Konishi , E. Akiyama , Y. Matsuzawa , et al., “Prognostic Impact of Muscle and Fat Mass in Patients With Heart Failure,” Journal of Cachexia, Sarcopenia and Muscle 12 (2021): 568–576. 33939328 10.1002/jcsm.12702 PMC8200420

[19] M. Konishi , N. Kagiyama , K. Kamiya , et al., “Impact of Sarcopenia on Prognosis in Patients With Heart Failure With Reduced and Preserved Ejection Fraction,” European Journal of Preventive Cardiology 28 (2021): 1022–1029. 33624112 10.1093/eurjpc/zwaa117

[20] R. Sato , M. Vatic , G. W. Peixoto da Fonseca , S. D. Anker , and S. von Haehling , “Biological Basis and Treatment of Frailty and Sarcopenia,” Cardiovascular Research 120 (2024): 982–998. 38828887 10.1093/cvr/cvae073

[21] S. von Haehling , M. Lainscak , W. Doehner , et al., “Diabetes Mellitus, Cachexia and Obesity in Heart Failure: Rationale and Design of the Studies Investigating Co‐Morbidities Aggravating Heart Failure (SICA‐HF),” Journal of Cachexia, Sarcopenia and Muscle 1 (2010): 187–194. 21475696 10.1007/s13539-010-0013-3 PMC3060647

[22] S. Fülster , M. Tacke , A. Sandek , et al., “Muscle Wasting in Patients With Chronic Heart Failure: Results From the Studies Investigating Co‐Morbidities Aggravating Heart Failure (SICA‐HF),” European Heart Journal 34 (2013): 512–519. 23178647 10.1093/eurheartj/ehs381

[23] R. N. Baumgartner , K. M. Koehler , D. Gallagher , et al., “Epidemiology of Sarcopenia Among the Elderly in New Mexico,” American Journal of Epidemiology 147 (1998): 755–763. 9554417 10.1093/oxfordjournals.aje.a009520

[24] W. J. Evans , J. E. Morley , J. Argilés , et al., “Cachexia: A New Definition,” Clinical Nutrition 27 (2008): 793–799. 18718696 10.1016/j.clnu.2008.06.013

[25] J. M. Guralnik , E. M. Simonsick , L. Ferrucci , et al., “A Short Physical Performance Battery Assessing Lower Extremity Function: Association With Self‐Reported Disability and Prediction of Mortality and Nursing Home Admission,” Journal of Gerontology 49 (1994): M85–M94. 8126356 10.1093/geronj/49.2.m85

[26] R. A. Bruce , J. R. Blackmon , J. W. Jones , and G. Strait , “Exercising Testing in Adult Normal Subjects and Cardiac Patients,” Pediatrics 32 (1963): 742–756. 14070531

[27] J. Naughton , G. Sevelius , and B. Balke , “Physiological Responses of Normal and Pathological Subjects to a Modified Work Capacity Test,” Journal of Sports Medicine and Physical Fitness 3 (1963): 201–207. 14099069

[28] G. F. Fletcher , P. A. Ades , P. Kligfield , et al., “Exercise Standards for Testing and Training: A Scientific Statement From the American Heart Association,” Circulation 128 (2013): 873–934. 23877260 10.1161/CIR.0b013e31829b5b44

[29] D. S. Schade , L. Shey , and R. P. Eaton , “Cholesterol Review: A Metabolically Important Molecule,” Endocrine Practice: Official Journal of the American College of Endocrinology and the American Association of Clinical Endocrinologists 26 (2020): 1514–1523. 33471744 10.4158/EP-2020-0347

[30] M. Wendel , R. Paul , and A. R. Heller , “Lipoproteins in Inflammation and Sepsis. II. Clinical Aspects,” Intensive Care Medicine 33 (2007): 25–35. 17093984 10.1007/s00134-006-0433-x

[31] C. S. Mermelstein , D. M. Portilho , R. B. Medeiros , et al., “Cholesterol Depletion by Methyl‐β‐Cyclodextrin Enhances Myoblast Fusion and Induces the Formation of Myotubes With Disorganized Nuclei,” Cell and Tissue Research 319 (2005): 289–297. 15549398 10.1007/s00441-004-1004-5

[32] J. Vega‐Moreno , A. Tirado‐Cortes , R. Álvarez , C. Irles , J. Mas‐Oliva , and A. Ortega , “Cholesterol Depletion Uncouples β‐Dystroglycans From Discrete Sarcolemmal Domains, Reducing the Mechanical Activity of Skeletal Muscle,” Cellular Physiology and Biochemistry: International Journal of Experimental Cellular Physiology, Biochemistry, and Pharmacology 29 (2012): 905–918. 22613990 10.1159/000186933

[33] C. Goossens , R. Weckx , S. Derde , et al., “Altered Cholesterol Homeostasis in Critical Illness‐Induced Muscle Weakness: Effect of Exogenous 3‐Hydroxybutyrate,” Critical Care (London, England) 25 (2021): 252. 34274000 10.1186/s13054-021-03688-1 PMC8285799

[34] A. Draeger , K. Monastyrskaya , M. Mohaupt , et al., “Statin Therapy Induces Ultrastructural Damage in Skeletal Muscle in Patients Without Myalgia,” Journal of Pathology 210 (2006): 94–102. 16799920 10.1002/path.2018

[35] R. W. Sherwin , D. N. Wentworth , J. A. Cutler , S. B. Hulley , L. H. Kuller , and J. Stamler , “Serum Cholesterol Levels and Cancer Mortality in 361,662 Men Screened for the Multiple Risk Factor Intervention Trial,” Journal of the American Medical Association 257 (1987): 943–948. 3806876

[36] D. A. Hofmaenner , P. Arina , A. Kleyman , et al., “Association Between Hypocholesterolemia and Mortality in Critically Ill Patients With Sepsis: A Systematic Review and Meta‐Analysis,” Critical Care Explorations 5 (2023): e0860. 36751516 10.1097/CCE.0000000000000860 PMC9894355

[37] S. Bodin , H. Tronchère , and B. Payrastre , “Lipid Rafts Are Critical Membrane Domains in Blood Platelet Activation Processes,” Biochimica et Biophysica Acta 1610 (2003): 247–257. 12648778 10.1016/s0005-2736(03)00022-1

[38] W. E. Alborn , G. Cao , H. E. Careskey , et al., “Serum Proprotein Convertase Subtilisin Kexin Type 9 Is Correlated Directly With Serum LDL Cholesterol,” Clinical Chemistry 53 (2007): 1814–1819. 17702855 10.1373/clinchem.2007.091280

[39] Z. Qi , L. Hu , J. Zhang , et al., “PCSK9 (Proprotein Convertase Subtilisin/Kexin 9) Enhances Platelet Activation, Thrombosis, and Myocardial Infarct Expansion by Binding to Platelet CD36,” Circulation 143 (2021): 45–61. 32988222 10.1161/CIRCULATIONAHA.120.046290

[40] C. Ma , M. E. Gurol , Z. Huang , et al., “Low‐Density Lipoprotein Cholesterol and Risk of Intracerebral Hemorrhage: A Prospective Study,” Neurology 93 (2019): e445–e457. 31266905 10.1212/WNL.0000000000007853 PMC6693427

[41] Q. Yang , D. Sun , C. Pei , et al., “LDL Cholesterol Levels and In‐Hospital Bleeding in Patients on High‐Intensity Antithrombotic Therapy: Findings From the CCC‐ACS Project,” European Heart Journal 42 (2021): 3175–3186. 34347859 10.1093/eurheartj/ehab418

[42] M. Klajda , B. Trachtenberg , R. Araujo , et al., “Pre‐Operative Sarcopenia Is Predictive of Recurrent Gastrointestinal Bleeding on Left Ventricular Assist Device Support: A Multicenter Analysis,” Journal of Heart and Lung Transplantation: The Official Publication of the International Society for Heart Transplantation 41 (2022): 757–762. 35105490 10.1016/j.healun.2022.01.004

[43] J. H. Butt , M. C. Petrie , P. S. Jhund , et al., “Anthropometric Measures and Adverse Outcomes in Heart Failure With Reduced Ejection Fraction: Revisiting the Obesity Paradox,” European Heart Journal 44 (2023): 1136–1153. 36944496 10.1093/eurheartj/ehad083 PMC10111968

[44] R. Sato and S. von Haehling , “Revisiting the Obesity Paradox in Heart Failure: What Is the Best Anthropometric Index to Gauge Obesity?,” European Heart Journal 44 (2023): 1154–1156. 36944505 10.1093/eurheartj/ehad079

[45] R. Sato and S. von Haehling , “Targeting Obesity for Therapeutic Intervention in Heart Failure Patients,” Expert Review of Cardiovascular Therapy 22 (2024): 217–230. 38864827 10.1080/14779072.2024.2363395

[46] M. Park and G. Sweeney , “Direct Effects of Adipokines on the Heart: Focus on Adiponectin,” Heart Failure Reviews 18 (2013): 631–644. 22893246 10.1007/s10741-012-9337-8

[47] P. P. Gupta , G. C. Fonarow , and T. B. Horwich , “Obesity and the Obesity Paradox in Heart Failure,” Canadian Journal of Cardiology 31 (2015): 195–202. 25661554 10.1016/j.cjca.2014.08.004

[48] J. Davignon , “Beneficial Cardiovascular Pleiotropic Effects of Statins,” Circulation 109 (2004): Iii39–Iii43. 15198965 10.1161/01.CIR.0000131517.20177.5a

[49] O. F. Kuzu , M. A. Noory , and G. P. Robertson , “The Role of Cholesterol in Cancer,” Cancer Research 76 (2016): 2063–2070. 27197250 10.1158/0008-5472.CAN-15-2613 PMC5813477

[50] P. Zhou , B. Li , B. Liu , T. Chen , and J. Xiao , “Prognostic Role of Serum Total Cholesterol and High‐Density Lipoprotein Cholesterol in Cancer Survivors: A Systematic Review and Meta‐Analysis,” Clinica Chimica Acta; International Journal of Clinical Chemistry 477 (2018): 94–104. 29223765 10.1016/j.cca.2017.11.039

[51] G. Fonseca , J. Farkas , E. Dora , S. von Haehling , and M. Lainscak , “Cancer Cachexia and Related Metabolic Dysfunction,” International Journal of Molecular Sciences 21, no. 7 (2020): 2321. 32230855 10.3390/ijms21072321 PMC7177950

[52] R. Sato , G. W. P. da Fonseca , W. das Neves , and S. von Haehling , “Mechanisms and Pharmacotherapy of Cancer Cachexia‐Associated Anorexia,” Pharmacology Research & Perspectives 13 (2025): e70031. 39776294 10.1002/prp2.70031 PMC11707257

[53] C. Thongprayoon , W. Cheungpasitporn , and K. Kashani , “Serum Creatinine Level, a Surrogate of Muscle Mass, Predicts Mortality in Critically Ill Patients,” Journal of Thoracic Disease 8 (2016): E305–E311. 27162688 10.21037/jtd.2016.03.62 PMC4842835

[54] D. Groothof , N. B. N. Shehab , N. S. Erler , et al., “Creatinine, Cystatin C, Muscle Mass, and Mortality: Findings From a Primary and Replication Population‐Based Cohort,” Journal of Cachexia, Sarcopenia and Muscle 15 (2024): 1528–1538. 38898741 10.1002/jcsm.13511 PMC11294032

[55] B. A. Borlaug , M. D. Jensen , D. W. Kitzman , C. S. P. Lam , M. Obokata , and O. J. Rider , “Obesity and Heart Failure With Preserved Ejection Fraction: New Insights and Pathophysiological Targets,” Cardiovascular Research 118 (2023): 3434–3450. 35880317 10.1093/cvr/cvac120 PMC10202444

[56] Y. Miura , S. Higuchi , K. Matsushita , et al., “Clinical Impact of Visceral‐to‐Subcutaneous Fat Ratio in Patients With Acute Aortic Dissection,” PLoS ONE 14 (2019): e0226642. 31869368 10.1371/journal.pone.0226642 PMC6927613

[57] R. Sato , K. Okada , E. Akiyama , et al., “Impact of Sarcopenic Obesity on Long‐Term Clinical Outcomes After ST‐Segment Elevation Myocardial Infarction,” Atherosclerosis 335 (2021): 135–141. 34517989 10.1016/j.atherosclerosis.2021.08.038

