# Supplementary material for: Skeletal Muscle Mass Modifies the Prognostic Impact of LDL Cholesterol in Chronic Heart Failure
Source: J Cachexia Sarcopenia Muscle. 2026 Jan 16;17(1):e70168. doi: 10.1002/jcsm.70168 (PMC12809719; doi:10.1002/jcsm.70168)
Supplement: Supplementary file 1 — Table S1: Baseline characteristics of patients by low and high LDL‐C levels. Table S2: Factors associated with all‐cause mortality in the low ASMI group. Table S3: Baseline characteristics of patients stratified by HF phenotype. Table S4: Baseline characteristics of patients stratified by statin use. [file JCSM-17-e70168-s003.docx]

**Supplementary Table**

**Supplementary Table 1. Baseline characteristics of patients by low and high LDL-C levels**

| **Variables** | **Overall**  **(*n*=241)** | **Low LDL-C (*n*=121)** | **High LDL-C (*n*=120)** | ***p*-value** |
| --- | --- | --- | --- | --- |
| Age, years | 68 ± 11 | 68 ± 11 | 67 ± 11 | 0.85 |
| Male sex, *n* (%) | 192 (79.7) | 107 (88.4) | 85 (70.8) | 0.0007 |
| BMI, kg/m^2^ | 29.0 ± 5.1 | 29.0 ± 5.4 | 29.0 ± 4.9 | 0.87 |
| NYHA class | 2.3 ± 0.6 | 2.4 ± 0.6 | 2.3 ± 0.6 | 0.20 |
| LVEF, % | 39 ± 13 | 37 ± 13 | 40 ± 13 | 0.23 |
| HFpEF, *n* (%) | 75 (31.1) | 32 (26.4) | 43 (35.8) | 0.12 |
| **Comorbidities, *n* (%)** |  |  |  |  |
| Hypertension | 193 (81.1) | 102 (85.0) | 91 (77.1) | 0.12 |
| Diabetes mellitus | 92 (38.8) | 55 (46.2) | 37 (31.4) | 0.02 |
| Current smoking | 29 (12.0) | 14 (11.6) | 15 (12.5) | 0.82 |
| CAD | 140 (58.3) | 85 (70.3) | 55 (46.2) | 0.0002 |
| Atrial fibrillation | 91 (37.8) | 48 (39.7) | 43 (35.8) | 0.54 |
| Anaemia | 76 (31.5) | 41 (33.9) | 35 (29.2) | 0.43 |
| Muscle wasting | 45 (18.7) | 25 (20.7) | 20 (16.7) | 0.81 |
| Cardiac cachexia | 56 (23.3) | 28 (23.3) | 28 (23.3) | 1.00 |
| **Laboratory data** |  |  |  |  |
| High-sensitivity CRP, mg/L | 1.7 [1.0-3.4] | 1.6 [0.8-3.3] | 2.1 [1.1-3.6] | 0.09 |
| Creatinine, mg/dL | 1.2 ± 0.4 | 1.3 ± 0.5 | 1.1 ± 0.4 | 0.04 |
| Haemoglobin, g/dL | 13.3 [12.4-14.5] | 13.3 [12.6-14.4] | 13.3 [12.4-14.5] | 0.58 |
| Albumin, g/L | 37 [35-39] | 37 [35-40] | 37 [35-39] | 0.90 |
| NT-proBNP, pg/mL | 586[224-1412] | 625 [204-1283] | 542 [231-1570] | 0.86 |
| HDL-C, mg/dL | 45 [37-57] | 43 [35-56] | 47 [38-57] | 0.16 |
| LDL-C, mg/dL | 93 [70-122] | 70 [59-80] | 122 [107-141] | <0.0001 |
| TG, mg/dL | 110 [83-169] | 104 [75-144] | 117 [90-179] | 0.01 |
| **Skeletal muscle and fat** |  |  |  |  |
| Handgrip strength, kg | 38 ± 12 | 38 ± 11 | 37 ± 12 | 0.17 |
| Quadriceps strength, kg | 39 ± 13 | 40 ± 13 | 39 ± 14 | 0.50 |
| ASMI, kg/m^2^ | 7.8 ± 1.1 | 7.9 ± 1.2 | 7.7 ± 1.1 | 0.20 |
| Fat mass, kg/m^2^ | 9.7 ± 3.6 | 9.4 ± 3.5 | 10.1 ± 3.8 | 0.12 |
| **Functional capacity** |  |  |  |  |
| 6-minute walk distance, m | 422 ± 138 | 413 ± 145 | 430 ± 131 | 0.33 |
| SPPB | 11 [9-12] | 11 [9-12] | 11 [10-12] | 0.28 |
| PeakVO_2_, ml/min/kg | 16.9 ± 5.0 | 16.3 ± 4.9 | 17.4 ± 5.0 | 0.11 |
| **Medication, *n* (%)** |  |  |  |  |
| ACE-I or ARB | 226 (93.8) | 116 (95.9) | 110 (91.7) | 0.18 |
| β-blockers | 220 (91.3) | 117 (96.7) | 103 (85.8) | 0.003 |
| MRA | 112 (46.5) | 61 (50.4) | 51 (42.5) | 0.22 |
| Loop diuretics | 131 (54.6) | 62 (51.2) | 69 (58.0) | 0.29 |
| Statins | 165 (68.5) | 109 (90.1) | 56 (46.7) | <0.0001 |
| Lipid-lowering agents | 171 (71.3) | 109 (90.1) | 62 (52.1) | <0.0001 |
| Aspirin | 166 (68.9) | 99 (81.8) | 67 (55.8) | <0.0001 |
| Oral anticoagulants | 82 (34.3) | 41 (34.2) | 41 (34.5) | 0.96 |

The values are numbers (percentages), means ± standard deviations, or medians [25^th^–75^th^ percentile range]. ACE-I, angiotensin-converting enzyme inhibitor; ARB, angiotensin II receptor blocker; ASMI; appendicular skeletal muscle mass index; BMI, body mass index, aspartate transaminase; CAD, coronary artery disease; CRP, C-reactive protein; HDL-C, high-density lipoprotein cholesterol; HFpEF, heart failure with preserved ejection fraction; LDL-C, low-density lipoprotein cholesterol; LVEF, left ventricular ejection fraction; MRA, mineral corticoid receptor antagonist; NT-proBNP, N-terminal pro-B-type natriuretic peptide; NYHA, New York Heart Association; SPPB, Short Physical Performance Battery; TG, triglyceride; VO_2_, oxygen consumption.

**Supplementary Table 2. Factors associated with all-cause mortality in the low ASMI group**

| **Variables** | **All-cause mortality** | | |
| --- | --- | --- | --- |
|  | **HR** | **95 % CI** | ***P*-value** |
| Age, per 1 year | 1.03 | 1.00-1.06 | 0.04 |
| Male sex | 2.30 | 1.04-5.07 | 0.04 |
| BMI, per 1 kg/m² | 0.96 | 0.90-1.02 | 0.17 |
| NYHA class, per 1 class increase | 2.00 | 1.28-3.16 | 0.003 |
| LVEF, per 10% increase | 0.64 | 0.50-0.82 | 0.0006 |
| Hypertension (present) | 1.71 | 0.88-3.32 | 0.11 |
| Diabetes mellitus (present) | 1.75 | 1.02-2.99 | 0.04 |
| Current smoking (present) | 1.73 | 0.85-3.54 | 0.13 |
| CAD (present) | 2.22 | 1.17-4.22 | 0.01 |
| Atrial fibrillation (present) | 1.82 | 1.08-3.08 | 0.02 |
| Anaemia (present) | 1.60 | 0.95-2.68 | 0.08 |
| Muscle wasting (present) | 1.52 | 0.90-2.57 | 0.12 |
| Cardiac cachexia (present) | 1.40 | 0.89-2.20 | 0.14 |
| Hs-CRP, per 1 mg/L increase | 1.12 | 0.95-1.32 | 0.16 |
| Creatinine, per 0.1 mg/dL increase | 1.06 | 1.02-1.10 | 0.003 |
| Haemoglobin, per 1 g/dL increase | 0.78 | 0.64-0.96 | 0.02 |
| Albumin, per 1 g/L increase | 0.96 | 0.91-1.02 | 0.15 |
| NT-proBNP, per 1 SD increase | 1.36 | 1.12-1.58 | 0.0003 |
| HDL-C, per 1 SD increase | 0.76 | 0.53-1.04 | 0.11 |
| LDL-C, per 1 SD increase | 0.61 | 0.43-0.82 | 0.003 |
| TG, per 1SD increase | 0.83 | 0.58-1.10 | 0.26 |
| Handgrip strength, per 1 kg increase | 0.99 | 0.97-1.02 | 0.71 |
| Quadriceps strength, per 1 kg increase | 0.97 | 0.95-0.99 | 0.04 |
| ASMI, per 1 kg/m² increase | 1.03 | 0.72-1.51 | 0.89 |
| Fat mass, per 1 kg/m² increase | 0.91 | 0.84-0.98 | 0.02 |
| 6-minute walk distance, per 10 m increase | 0.96 | 0.94-0.98 | 0.0001 |
| SPPB, per 1 point increase | 0.83 | 0.76-0.91 | <0.0001 |
| PeakVO2, per 1 mL/min/kg increase | 0.87 | 0.81-0.94 | <0.0001 |
| ACE-I/ARB (present) | 0.91 | 0.36-2.27 | 0.84 |
| β-blockers (present) | 1.40 | 0.50-3.86 | 0.50 |
| MRA (present) | 1.51 | 0.90-2.55 | 0.12 |
| Loop diuretics (present) | 1.90 | 1.12-3.25 | 0.02 |
| Statins (present) | 1.16 | 0.66-2.05 | 0.61 |
| Lipid-lowering agents (present) | 0.93 | 0.52-1.67 | 0.82 |
| Aspirin (present) | 2.03 | 1.07-3.86 | 0.03 |
| Oral anticoagulants (present) | 1.28 | 0.76-2.18 | 0.36 |

ACE-I, angiotensin-converting enzyme inhibitor; ARB, angiotensin II receptor blocker; ASMI; appendicular skeletal muscle mass index; BMI, body mass index, aspartate transaminase; CAD, coronary artery disease; CI, confidence interval; CRP, C-reactive protein; HDL-C, high-density lipoprotein cholesterol; HR, hazard ratio; LDL-C, low-density lipoprotein cholesterol; LVEF, left ventricular ejection fraction; MRA, mineral corticoid receptor antagonist; NT-proBNP, N-terminal pro-B-type natriuretic peptide; NYHA, New York Heart Association; SD, standard deviation; SPPB, Short Physical Performance Battery; TG, triglyceride; VO_2_, oxygen consumption.

**Supplementary Table 3. Baseline characteristics of patients stratified by HF phenotype**

| **Variables** | **Overall**  **(*n*=241)** | **HFpEF**  **(*n*=75)** | **HFrEF**  **(*n*=166)** | ***p*-value** |
| --- | --- | --- | --- | --- |
| Age, years | 68 ± 11 | 69 ± 11 | 67 ± 10 | 0.03 |
| Male sex, *n* (%) | 192 (79.7) | 51 (68.0) | 141 (84.9) | 0.003 |
| BMI, kg/m^2^ | 29.0 ± 5.1 | 30.5 ± 5.0 | 28.3 ± 5.0 | 0.003 |
| NYHA class | 2.3 ± 0.6 | 2.2 ± 0.7 | 2.4 ± 0.6 | 0.02 |
| LVEF, % | 39 ± 13 | 55 ± 6 | 31 ± 7 | <0.0001 |
| **Comorbidities, *n* (%)** |  |  |  |  |
| Hypertension | 193 (81.1) | 66 (90.4) | 127 (77.0) | 0.01 |
| Diabetes mellitus | 92 (38.8) | 31 (43.1) | 61 (37.0) | 0.38 |
| Current smoking | 29 (12.0) | 7 (9.3) | 22 (13.3) | 0.39 |
| CAD | 140 (58.3) | 22 (29.3) | 118 (71.5) | <0.0001 |
| Atrial fibrillation | 91 (37.8) | 20 (26.7) | 71 (42.8) | 0.02 |
| Anaemia | 76 (31.5) | 19 (25.3) | 57 (34.3) | 0.16 |
| Muscle wasting | 45 (18.7) | 8 (10.7) | 37 (22.3) | 0.03 |
| Cardiac cachexia | 56 (23.3) | 10 (13.5) | 46 (27.7) | 0.02 |
| **Laboratory data** |  |  |  |  |
| High-sensitivity CRP, mg/L | 1.7 [1.0-3.4] | 1.8 [0.9-3.6] | 1.7 [1.0-3.3] | 0.72 |
| Creatinine, mg/dL | 1.2 ± 0.4 | 1.1 ± 0.3 | 1.2 ± 0.5 | 0.01 |
| Haemoglobin, g/dL | 13.3 [12.4-14.5] | 13.5 [12.6-14.5] | 13.3 [12.3-14.4] | 0.82 |
| Albumin, g/L | 37 [35-39] | 37 [35-40] | 37 [35-39] | 0.57 |
| NT-proBNP, pg/mL | 586[224-1412] | 217 [111-439] | 915 [436-2398] | <0.0001 |
| HDL-C, mg/dL | 45 [37-57] | 50 [38-61] | 44 [37-53] | 0.02 |
| LDL-C, mg/dL | 93 [70-122] | 105 [74-128] | 89 [66-114] | 0.03 |
| TG, mg/dL | 110 [83-169] | 112 [79-190] | 110 [83-163] | 0.73 |
| **Skeletal muscle and fat** |  |  |  |  |
| Handgrip strength, kg | 38 ± 12 | 37 ± 12 | 38 ± 12 | 0.86 |
| Quadriceps strength, kg | 39 ± 13 | 40 ± 13 | 39 ± 13 | 0.46 |
| ASMI, kg/m^2^ | 7.8 ± 1.1 | 7.9 ± 1.1 | 7.8 ± 1.1 | 0.51 |
| Fat mass, kg/m^2^ | 9.7 ± 3.6 | 11.1 ± 3.8 | 9.1 ± 3.4 | <0.0001 |
| **Functional capacity** |  |  |  |  |
| 6-minute walk distance, m | 422 ± 138 | 439 ± 129 | 413 ± 142 | 0.22 |
| SPPB | 11 [9-12] | 11 [10-12] | 11 [9-12] | 0.36 |
| PeakVO_2_, ml/min/kg | 16.9 ± 5.0 | 18.2 ± 5.1 | 16.2 ± 4.8 | 0.007 |
| **Medication, *n* (%)** |  |  |  |  |
| ACE-I or ARB | 226 (93.8) | 67 (89.3) | 159 (95.8) | 0.06 |
| β-blockers | 220 (91.3) | 62 (82.7) | 158 (95.2) | 0.001 |
| MRA | 112 (46.5) | 15 (20.0) | 97 (58.4) | <0.0001 |
| Loop diuretics | 131 (54.6) | 25 (33.3) | 106 (64.2) | <0.0001 |
| Statins | 165 (68.5) | 45 (60.0) | 120 (72.3) | 0.06 |
| Lipid-lowering agents | 171 (71.3) | 49 (65.3) | 122 (73.9) | 0.17 |
| Aspirin | 166 (68.9) | 46 (61.3) | 120 (72.3) | 0.09 |
| Oral anticoagulants | 82 (34.3) | 16 (21.6) | 66 (40.0) | 0.006 |

The values are numbers (percentages), means ± standard deviations, or medians [25^th^–75^th^ percentile range]. ACE-I, angiotensin-converting enzyme inhibitor; ARB, angiotensin II receptor blocker; ASMI; appendicular skeletal muscle mass index; BMI, body mass index, aspartate transaminase; CAD, coronary artery disease; CRP, C-reactive protein; HDL-C, high-density lipoprotein cholesterol; HFpEF, heart failure with preserved ejection fraction: HFrEF, heart failure with reduced ejection fraction; LDL-C, low-density lipoprotein cholesterol; LVEF, left ventricular ejection fraction; MRA, mineral corticoid receptor antagonist; NT-proBNP, N-terminal pro-B-type natriuretic peptide; NYHA, New York Heart Association; SPPB, Short Physical Performance Battery; TG, triglyceride; VO_2_, oxygen consumption.

**Supplementary Table 4. Baseline characteristics of patients stratified by statin use**

| **Variables** | **Overall**  **(*n*=241)** | **Statin group**  **(*n*=165)** | **Non-statin group**  **(*n*=76)** | ***p*-value** |
| --- | --- | --- | --- | --- |
| Age, years | 68 ± 11 | 68 ± 10 | 68 ± 11 | 0.79 |
| Male sex, *n* (%) | 192 (79.7) | 140 (84.9) | 52 (68.4) | 0.003 |
| BMI, kg/m^2^ | 29.0 ± 5.1 | 29.6 ± 5.3 | 27.7 ± 4.6 | 0.01 |
| NYHA class | 2.3 ± 0.6 | 2.4 ± 0.6 | 2.2 ± 0.7 | 0.15 |
| LVEF, % | 39 ± 13 | 37 ± 13 | 41 ± 13 | 0.09 |
| HFpEF, *n* (%) | 75 (31.1) | 45 (27.3) | 30 (39.5) | 0.06 |
| **Comorbidities, *n* (%)** |  |  |  |  |
| Hypertension | 193 (81.1) | 140 (85.9) | 53 (70.7) | 0.005 |
| Diabetes mellitus | 92 (38.8) | 68 (42.0) | 24 (32.0) | 0.14 |
| Current smoking | 29 (12.0) | 21 (12.7) | 8 (10.5) | 0.63 |
| CAD | 140 (58.3) | 117 (71.3) | 23 (30.3) | <0.0001 |
| Atrial fibrillation | 91 (37.8) | 59 (35.8) | 32 (42.1) | 0.34 |
| Anaemia | 76 (31.5) | 45 (27.3) | 31 (40.8) | 0.04 |
| Muscle wasting | 45 (18.7) | 32 (19.4) | 13 (17.1) | 0.67 |
| Cardiac cachexia | 56 (23.3) | 37 (22.6) | 19 (25.0) | 0.68 |
| **Laboratory data** |  |  |  |  |
| High-sensitivity CRP, mg/L | 1.7 [1.0-3.4] | 1.6 [0.9-3.3] | 2.1 [1.2-3.5] | 0.20 |
| Creatinine, mg/dL | 1.2 ± 0.4 | 1.2 ± 0.4 | 1.2 ± 0.5 | 0.13 |
| Haemoglobin, g/dL | 13.3 [12.4-14.5] | 13.5 [12.8-14.5] | 13.0 [12.0-14.5] | 0.04 |
| Albumin, g/L | 37 [35-39] | 37 [35-39] | 37 [35-39] | 0.86 |
| NT-proBNP, pg/mL | 586[224-1412] | 555 [195-1311] | 603 [267-1572] | 0.45 |
| HDL-C, mg/dL | 45 [37-57] | 45 [36-54] | 46 [38-60] | 0.22 |
| LDL-C, mg/dL | 93 [70-122] | 80 [63-103] | 124 [104-154] | <0.0001 |
| TG, mg/dL | 110 [83-169] | 106 [80-166] | 118 [87-179] | 0.29 |
| **Skeletal muscle and fat** |  |  |  |  |
| Handgrip strength, kg | 38 ± 12 | 38 ± 11 | 36 ± 12 | 0.13 |
| Quadriceps strength, kg | 39 ± 13 | 40 ± 13 | 38 ± 14 | 0.40 |
| ASMI, kg/m^2^ | 7.8 ± 1.1 | 7.9 ± 1.2 | 7.6 ± 1.0 | 0.04 |
| Fat mass, kg/m^2^ | 9.7 ± 3.6 | 10.0 ± 3.7 | 9.2 ± 3.5 | 0.16 |
| **Functional capacity** |  |  |  |  |
| 6-minute walk distance, m | 422 ± 138 | 413 ± 138 | 440 ± 137 | 0.19 |
| SPPB | 11 [9-12] | 11 [10-12] | 11 [9-12] | 0.26 |
| PeakVO_2_, ml/min/kg | 16.9 ± 5.0 | 16.4 ± 4.7 | 17.8 ± 5.3 | 0.052 |
| **Medication, *n* (%)** |  |  |  |  |
| ACE-I or ARB | 226 (93.8) | 158 (95.8) | 68 (89.5) | 0.06 |
| β-blockers | 220 (91.3) | 157 (95.2) | 63 (82.9) | 0.002 |
| MRA | 112 (46.5) | 82 (49.7) | 30 (39.5) | 0.14 |
| Loop diuretics | 131 (54.6) | 94 (57.3) | 37 (48.7) | 0.21 |
| Statins | 165 (68.5) | 165 (100) | 0 (0) | <0.0001 |
| Lipid-lowering agents | 171 (71.3) | 165 (100) | 6 (8.0) | <0.0001 |
| Aspirin | 166 (68.9) | 130 (78.8) | 36 (47.4) | <0.0001 |
| Oral anticoagulants | 82 (34.3) | 56 (34.2) | 26 (34.7) | 0.94 |

The values are numbers (percentages), means ± standard deviations, or medians [25^th^–75^th^ percentile range]. ACE-I, angiotensin-converting enzyme inhibitor; ARB, angiotensin II receptor blocker; ASMI; appendicular skeletal muscle mass index; BMI, body mass index, aspartate transaminase; CAD, coronary artery disease; CRP, C-reactive protein; HDL-C, high-density lipoprotein cholesterol; HFpEF, heart failure with preserved ejection fraction; LDL-C, low-density lipoprotein cholesterol; LVEF, left ventricular ejection fraction; MRA, mineral corticoid receptor antagonist; NT-proBNP, N-terminal pro-B-type natriuretic peptide; NYHA, New York Heart Association; SPPB, Short Physical Performance Battery; TG, triglyceride; VO_2_, oxygen consumption.
