# Supplementary figures and images for: Skeletal Muscle Mass Modifies the Prognostic Impact of LDL Cholesterol in Chronic Heart Failure
Source: J Cachexia Sarcopenia Muscle. 2026 Jan 16;17(1):e70168. doi: 10.1002/jcsm.70168 (PMC12809719; doi:10.1002/jcsm.70168)

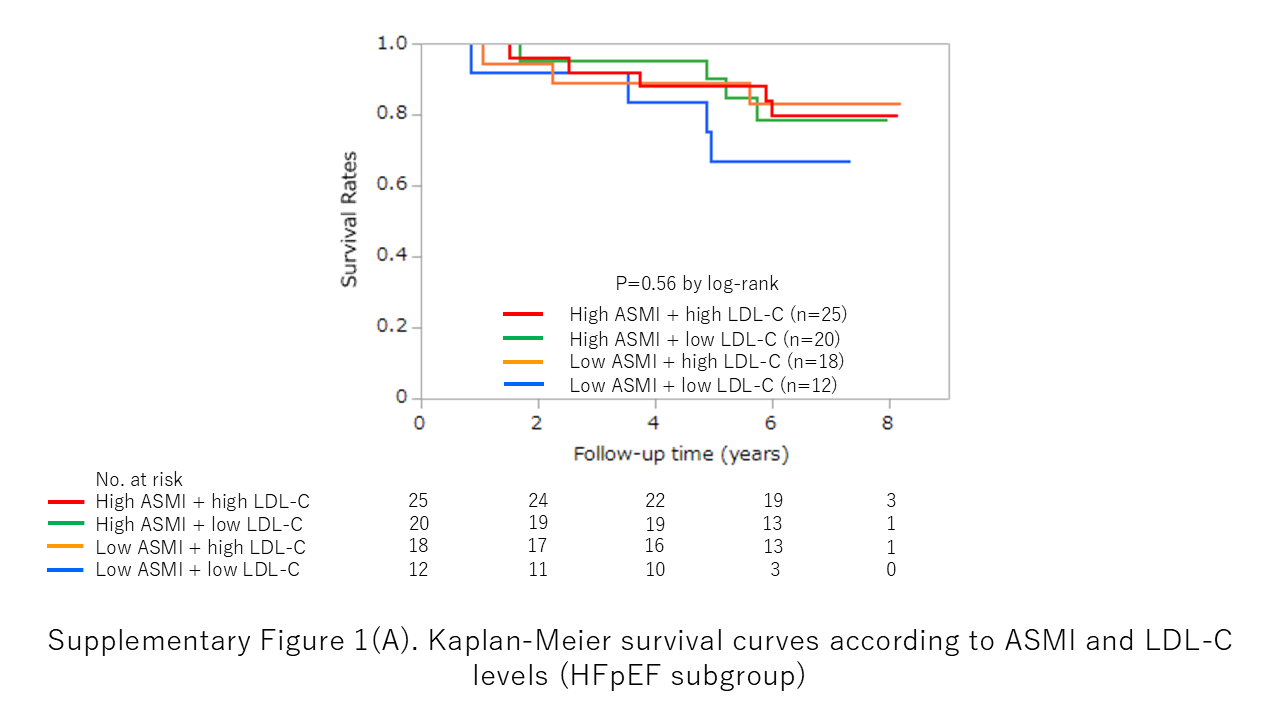

Supplement: Supplementary file 2 — Figure S1: Kaplan–Meier survival curves according to ASMI and LDL‐C levels. (A) HFpEF subgroup. (B) HFrEF subgroup. ASMI, appendicular skeletal muscle mass index; HFpEF, heart failure with preserved ejection fraction; HFrEF, heart failure with reduced ejection fraction; LDL‐C, low‐density lipoprotein cholesterol. Figure S2: Impact of LDL‐C levels (A) and ASMI (B) on all‐cause mortality in patients with HFpEF and HFrEF. ASMI, appendicular skeletal muscle mass index; HFpEF, heart failure with preserved ejection fraction; HFrEF, heart failure with reduced ejection fraction; LDL‐C, low‐density lipoprotein cholesterol. Figure S3: Impact of LDL‐C levels on all‐cause mortality according to the ASMI value in patients with HFrEF. ASMI, appendicular skeletal muscle mass index; HFrEF, heart failure with reduced ejection fraction; LDL‐C, low‐density lipoprotein cholesterol. Figure S4: Kaplan–Meier survival curves according to ASMI and LDL‐C levels. (A) Statin subgroup. (B) Nonstatin subgroup. ASMI, appendicular skeletal muscle mass index; LDL‐C, low‐density lipoprotein cholesterol. Figure S5: Impact of LDL‐C levels (A) and ASMI (B) on all‐cause mortality in patients with or without statin use. ASMI, appendicular skeletal muscle mass index; LDL‐C, low‐density lipoprotein cholesterol. Figure S6: Impact of LDL‐C levels on all‐cause mortality according to the ASMI value in the nonstatin subgroup. ASMI, appendicular skeletal muscle mass index; LDL‐C, low‐density lipoprotein cholesterol. Figure S7: Kaplan–Meier survival curves according to ASMI and LDL‐C levels (patients surviving > 2 years). ASMI, appendicular skeletal muscle mass index; LDL‐C, low‐density lipoprotein cholesterol. Figure S8: Impact of LDL‐C levels and ASMI on all‐cause mortality (patients surviving > 2 years). ASMI, appendicular skeletal muscle mass index; LDL‐C, low‐density lipoprotein cholesterol. Figure S9: Impact of LDL‐C levels on all‐cause mortality according to the ASMI value (patients surviving > 2 years). A [file JCSM-17-e70168-s001.zip › Supplementary Figure 1(A).TIF]

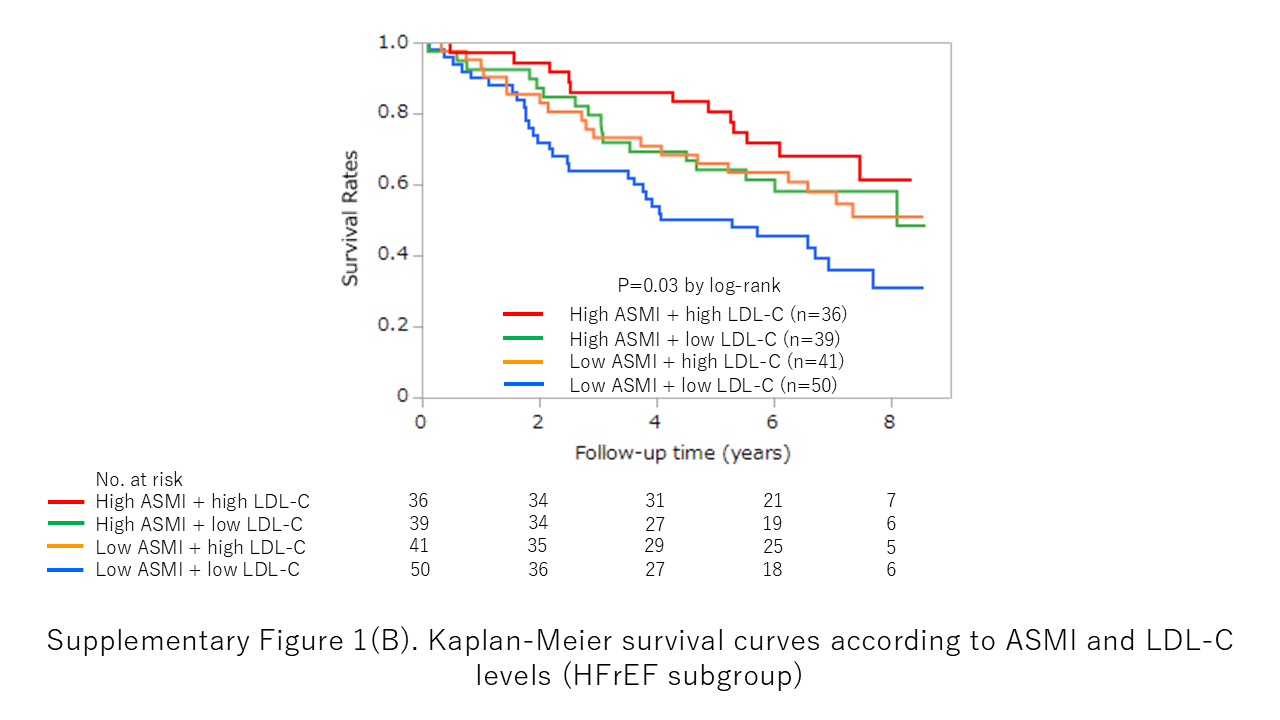

Supplement: Supplementary file 2 — Figure S1: Kaplan–Meier survival curves according to ASMI and LDL‐C levels. (A) HFpEF subgroup. (B) HFrEF subgroup. ASMI, appendicular skeletal muscle mass index; HFpEF, heart failure with preserved ejection fraction; HFrEF, heart failure with reduced ejection fraction; LDL‐C, low‐density lipoprotein cholesterol. Figure S2: Impact of LDL‐C levels (A) and ASMI (B) on all‐cause mortality in patients with HFpEF and HFrEF. ASMI, appendicular skeletal muscle mass index; HFpEF, heart failure with preserved ejection fraction; HFrEF, heart failure with reduced ejection fraction; LDL‐C, low‐density lipoprotein cholesterol. Figure S3: Impact of LDL‐C levels on all‐cause mortality according to the ASMI value in patients with HFrEF. ASMI, appendicular skeletal muscle mass index; HFrEF, heart failure with reduced ejection fraction; LDL‐C, low‐density lipoprotein cholesterol. Figure S4: Kaplan–Meier survival curves according to ASMI and LDL‐C levels. (A) Statin subgroup. (B) Nonstatin subgroup. ASMI, appendicular skeletal muscle mass index; LDL‐C, low‐density lipoprotein cholesterol. Figure S5: Impact of LDL‐C levels (A) and ASMI (B) on all‐cause mortality in patients with or without statin use. ASMI, appendicular skeletal muscle mass index; LDL‐C, low‐density lipoprotein cholesterol. Figure S6: Impact of LDL‐C levels on all‐cause mortality according to the ASMI value in the nonstatin subgroup. ASMI, appendicular skeletal muscle mass index; LDL‐C, low‐density lipoprotein cholesterol. Figure S7: Kaplan–Meier survival curves according to ASMI and LDL‐C levels (patients surviving > 2 years). ASMI, appendicular skeletal muscle mass index; LDL‐C, low‐density lipoprotein cholesterol. Figure S8: Impact of LDL‐C levels and ASMI on all‐cause mortality (patients surviving > 2 years). ASMI, appendicular skeletal muscle mass index; LDL‐C, low‐density lipoprotein cholesterol. Figure S9: Impact of LDL‐C levels on all‐cause mortality according to the ASMI value (patients surviving > 2 years). A [file JCSM-17-e70168-s001.zip › Supplementary Figure 1(B).TIF]

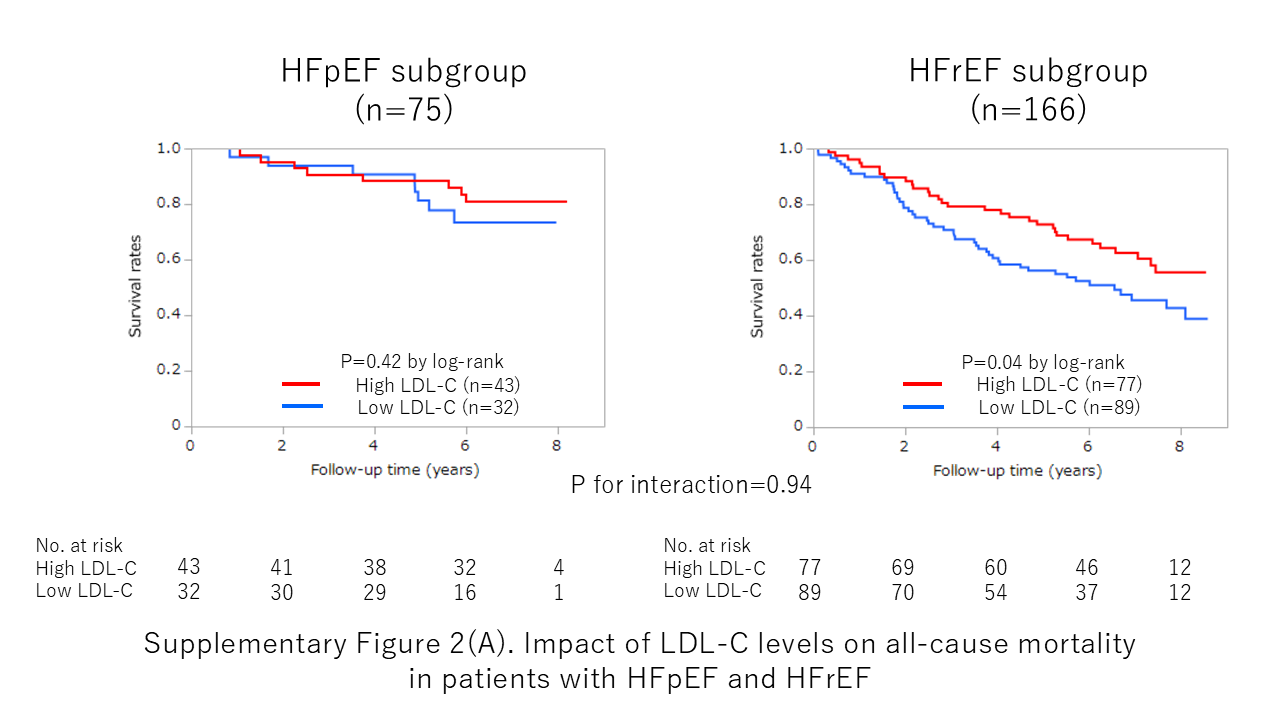

Supplement: Supplementary file 2 — Figure S1: Kaplan–Meier survival curves according to ASMI and LDL‐C levels. (A) HFpEF subgroup. (B) HFrEF subgroup. ASMI, appendicular skeletal muscle mass index; HFpEF, heart failure with preserved ejection fraction; HFrEF, heart failure with reduced ejection fraction; LDL‐C, low‐density lipoprotein cholesterol. Figure S2: Impact of LDL‐C levels (A) and ASMI (B) on all‐cause mortality in patients with HFpEF and HFrEF. ASMI, appendicular skeletal muscle mass index; HFpEF, heart failure with preserved ejection fraction; HFrEF, heart failure with reduced ejection fraction; LDL‐C, low‐density lipoprotein cholesterol. Figure S3: Impact of LDL‐C levels on all‐cause mortality according to the ASMI value in patients with HFrEF. ASMI, appendicular skeletal muscle mass index; HFrEF, heart failure with reduced ejection fraction; LDL‐C, low‐density lipoprotein cholesterol. Figure S4: Kaplan–Meier survival curves according to ASMI and LDL‐C levels. (A) Statin subgroup. (B) Nonstatin subgroup. ASMI, appendicular skeletal muscle mass index; LDL‐C, low‐density lipoprotein cholesterol. Figure S5: Impact of LDL‐C levels (A) and ASMI (B) on all‐cause mortality in patients with or without statin use. ASMI, appendicular skeletal muscle mass index; LDL‐C, low‐density lipoprotein cholesterol. Figure S6: Impact of LDL‐C levels on all‐cause mortality according to the ASMI value in the nonstatin subgroup. ASMI, appendicular skeletal muscle mass index; LDL‐C, low‐density lipoprotein cholesterol. Figure S7: Kaplan–Meier survival curves according to ASMI and LDL‐C levels (patients surviving > 2 years). ASMI, appendicular skeletal muscle mass index; LDL‐C, low‐density lipoprotein cholesterol. Figure S8: Impact of LDL‐C levels and ASMI on all‐cause mortality (patients surviving > 2 years). ASMI, appendicular skeletal muscle mass index; LDL‐C, low‐density lipoprotein cholesterol. Figure S9: Impact of LDL‐C levels on all‐cause mortality according to the ASMI value (patients surviving > 2 years). A [file JCSM-17-e70168-s001.zip › Supplementary Figure 2(A).TIF]

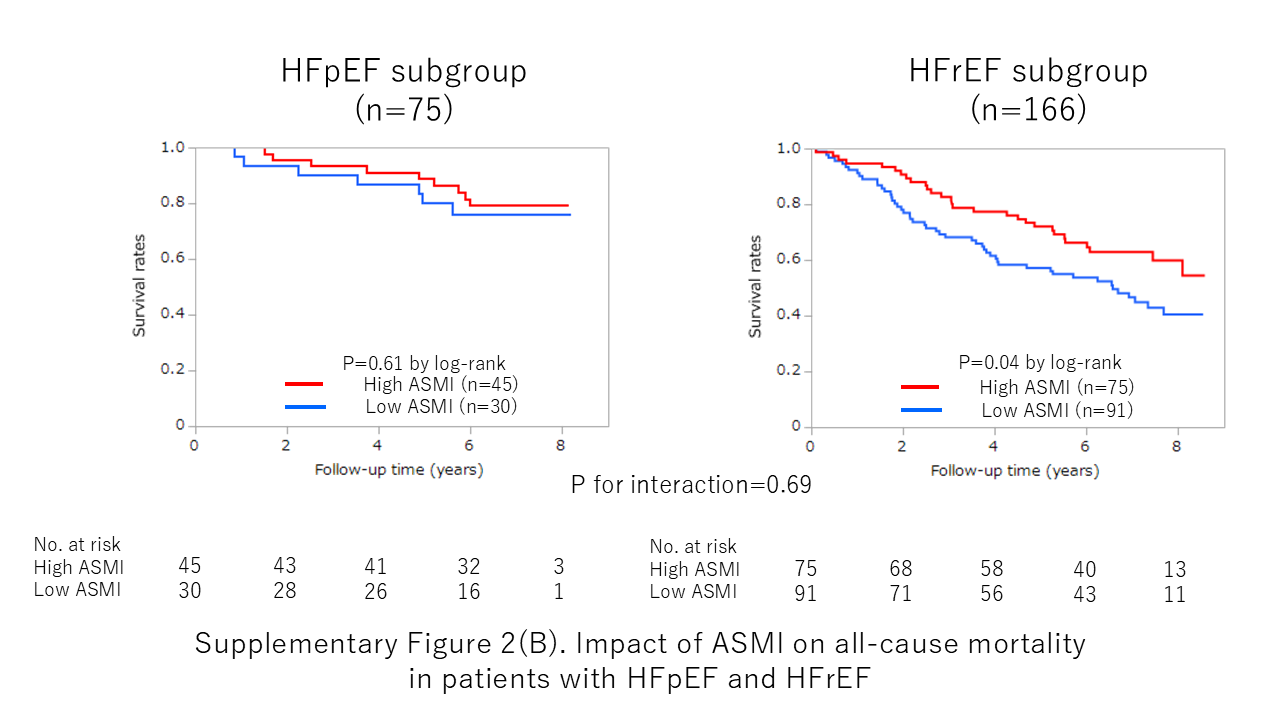

Supplement: Supplementary file 2 — Figure S1: Kaplan–Meier survival curves according to ASMI and LDL‐C levels. (A) HFpEF subgroup. (B) HFrEF subgroup. ASMI, appendicular skeletal muscle mass index; HFpEF, heart failure with preserved ejection fraction; HFrEF, heart failure with reduced ejection fraction; LDL‐C, low‐density lipoprotein cholesterol. Figure S2: Impact of LDL‐C levels (A) and ASMI (B) on all‐cause mortality in patients with HFpEF and HFrEF. ASMI, appendicular skeletal muscle mass index; HFpEF, heart failure with preserved ejection fraction; HFrEF, heart failure with reduced ejection fraction; LDL‐C, low‐density lipoprotein cholesterol. Figure S3: Impact of LDL‐C levels on all‐cause mortality according to the ASMI value in patients with HFrEF. ASMI, appendicular skeletal muscle mass index; HFrEF, heart failure with reduced ejection fraction; LDL‐C, low‐density lipoprotein cholesterol. Figure S4: Kaplan–Meier survival curves according to ASMI and LDL‐C levels. (A) Statin subgroup. (B) Nonstatin subgroup. ASMI, appendicular skeletal muscle mass index; LDL‐C, low‐density lipoprotein cholesterol. Figure S5: Impact of LDL‐C levels (A) and ASMI (B) on all‐cause mortality in patients with or without statin use. ASMI, appendicular skeletal muscle mass index; LDL‐C, low‐density lipoprotein cholesterol. Figure S6: Impact of LDL‐C levels on all‐cause mortality according to the ASMI value in the nonstatin subgroup. ASMI, appendicular skeletal muscle mass index; LDL‐C, low‐density lipoprotein cholesterol. Figure S7: Kaplan–Meier survival curves according to ASMI and LDL‐C levels (patients surviving > 2 years). ASMI, appendicular skeletal muscle mass index; LDL‐C, low‐density lipoprotein cholesterol. Figure S8: Impact of LDL‐C levels and ASMI on all‐cause mortality (patients surviving > 2 years). ASMI, appendicular skeletal muscle mass index; LDL‐C, low‐density lipoprotein cholesterol. Figure S9: Impact of LDL‐C levels on all‐cause mortality according to the ASMI value (patients surviving > 2 years). A [file JCSM-17-e70168-s001.zip › Supplementary Figure 2(B).TIF]

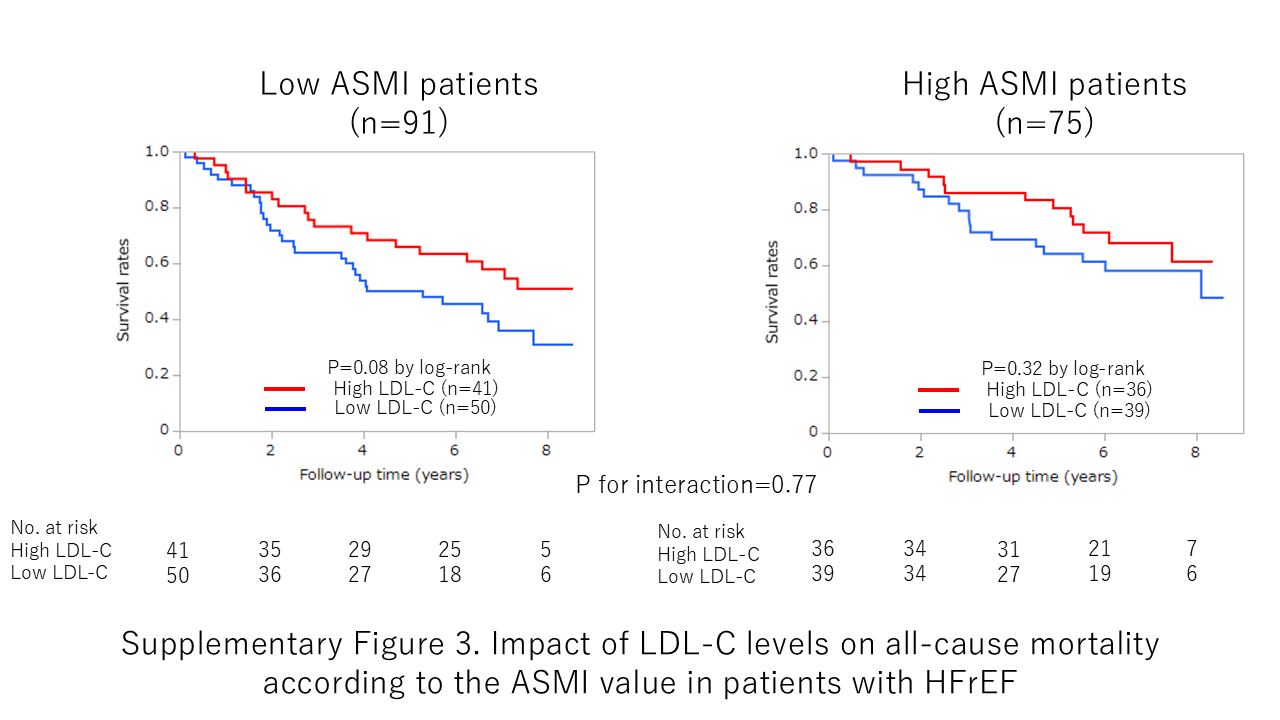

Supplement: Supplementary file 2 — Figure S1: Kaplan–Meier survival curves according to ASMI and LDL‐C levels. (A) HFpEF subgroup. (B) HFrEF subgroup. ASMI, appendicular skeletal muscle mass index; HFpEF, heart failure with preserved ejection fraction; HFrEF, heart failure with reduced ejection fraction; LDL‐C, low‐density lipoprotein cholesterol. Figure S2: Impact of LDL‐C levels (A) and ASMI (B) on all‐cause mortality in patients with HFpEF and HFrEF. ASMI, appendicular skeletal muscle mass index; HFpEF, heart failure with preserved ejection fraction; HFrEF, heart failure with reduced ejection fraction; LDL‐C, low‐density lipoprotein cholesterol. Figure S3: Impact of LDL‐C levels on all‐cause mortality according to the ASMI value in patients with HFrEF. ASMI, appendicular skeletal muscle mass index; HFrEF, heart failure with reduced ejection fraction; LDL‐C, low‐density lipoprotein cholesterol. Figure S4: Kaplan–Meier survival curves according to ASMI and LDL‐C levels. (A) Statin subgroup. (B) Nonstatin subgroup. ASMI, appendicular skeletal muscle mass index; LDL‐C, low‐density lipoprotein cholesterol. Figure S5: Impact of LDL‐C levels (A) and ASMI (B) on all‐cause mortality in patients with or without statin use. ASMI, appendicular skeletal muscle mass index; LDL‐C, low‐density lipoprotein cholesterol. Figure S6: Impact of LDL‐C levels on all‐cause mortality according to the ASMI value in the nonstatin subgroup. ASMI, appendicular skeletal muscle mass index; LDL‐C, low‐density lipoprotein cholesterol. Figure S7: Kaplan–Meier survival curves according to ASMI and LDL‐C levels (patients surviving > 2 years). ASMI, appendicular skeletal muscle mass index; LDL‐C, low‐density lipoprotein cholesterol. Figure S8: Impact of LDL‐C levels and ASMI on all‐cause mortality (patients surviving > 2 years). ASMI, appendicular skeletal muscle mass index; LDL‐C, low‐density lipoprotein cholesterol. Figure S9: Impact of LDL‐C levels on all‐cause mortality according to the ASMI value (patients surviving > 2 years). A [file JCSM-17-e70168-s001.zip › Supplementary Figure 3.TIF]

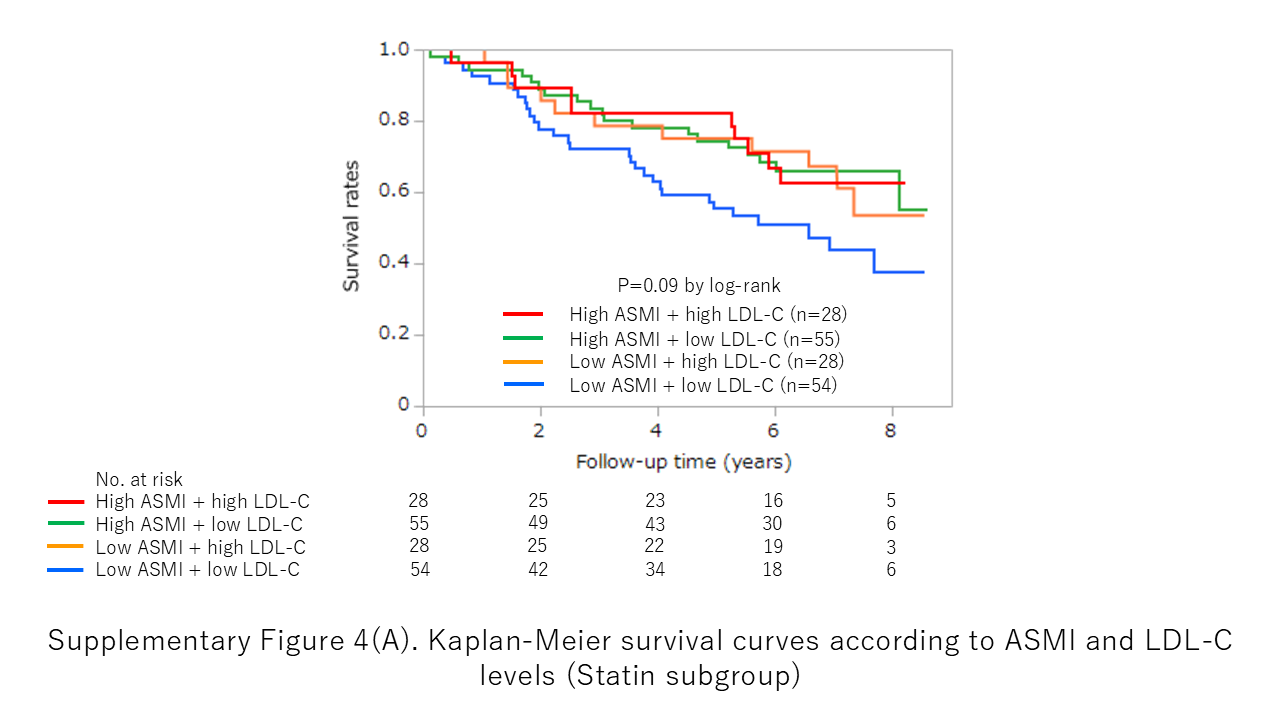

Supplement: Supplementary file 2 — Figure S1: Kaplan–Meier survival curves according to ASMI and LDL‐C levels. (A) HFpEF subgroup. (B) HFrEF subgroup. ASMI, appendicular skeletal muscle mass index; HFpEF, heart failure with preserved ejection fraction; HFrEF, heart failure with reduced ejection fraction; LDL‐C, low‐density lipoprotein cholesterol. Figure S2: Impact of LDL‐C levels (A) and ASMI (B) on all‐cause mortality in patients with HFpEF and HFrEF. ASMI, appendicular skeletal muscle mass index; HFpEF, heart failure with preserved ejection fraction; HFrEF, heart failure with reduced ejection fraction; LDL‐C, low‐density lipoprotein cholesterol. Figure S3: Impact of LDL‐C levels on all‐cause mortality according to the ASMI value in patients with HFrEF. ASMI, appendicular skeletal muscle mass index; HFrEF, heart failure with reduced ejection fraction; LDL‐C, low‐density lipoprotein cholesterol. Figure S4: Kaplan–Meier survival curves according to ASMI and LDL‐C levels. (A) Statin subgroup. (B) Nonstatin subgroup. ASMI, appendicular skeletal muscle mass index; LDL‐C, low‐density lipoprotein cholesterol. Figure S5: Impact of LDL‐C levels (A) and ASMI (B) on all‐cause mortality in patients with or without statin use. ASMI, appendicular skeletal muscle mass index; LDL‐C, low‐density lipoprotein cholesterol. Figure S6: Impact of LDL‐C levels on all‐cause mortality according to the ASMI value in the nonstatin subgroup. ASMI, appendicular skeletal muscle mass index; LDL‐C, low‐density lipoprotein cholesterol. Figure S7: Kaplan–Meier survival curves according to ASMI and LDL‐C levels (patients surviving > 2 years). ASMI, appendicular skeletal muscle mass index; LDL‐C, low‐density lipoprotein cholesterol. Figure S8: Impact of LDL‐C levels and ASMI on all‐cause mortality (patients surviving > 2 years). ASMI, appendicular skeletal muscle mass index; LDL‐C, low‐density lipoprotein cholesterol. Figure S9: Impact of LDL‐C levels on all‐cause mortality according to the ASMI value (patients surviving > 2 years). A [file JCSM-17-e70168-s001.zip › Supplementary Figure 4(A).TIF]

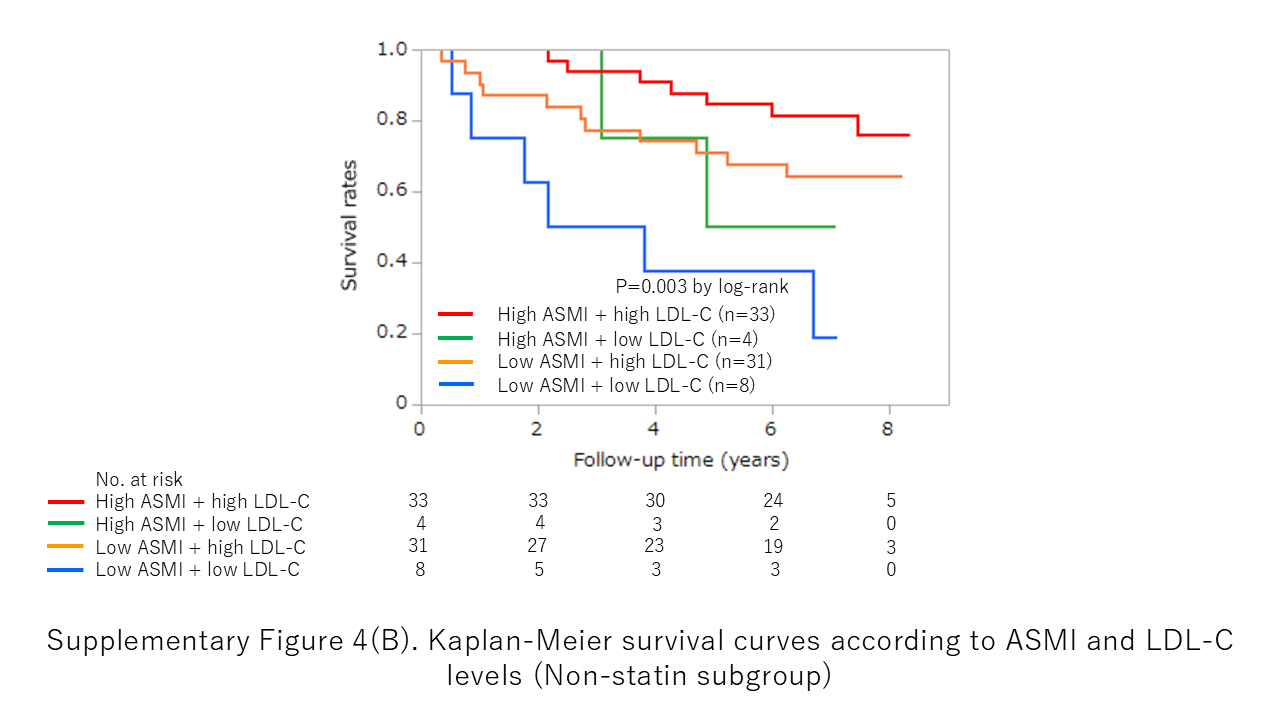

Supplement: Supplementary file 2 — Figure S1: Kaplan–Meier survival curves according to ASMI and LDL‐C levels. (A) HFpEF subgroup. (B) HFrEF subgroup. ASMI, appendicular skeletal muscle mass index; HFpEF, heart failure with preserved ejection fraction; HFrEF, heart failure with reduced ejection fraction; LDL‐C, low‐density lipoprotein cholesterol. Figure S2: Impact of LDL‐C levels (A) and ASMI (B) on all‐cause mortality in patients with HFpEF and HFrEF. ASMI, appendicular skeletal muscle mass index; HFpEF, heart failure with preserved ejection fraction; HFrEF, heart failure with reduced ejection fraction; LDL‐C, low‐density lipoprotein cholesterol. Figure S3: Impact of LDL‐C levels on all‐cause mortality according to the ASMI value in patients with HFrEF. ASMI, appendicular skeletal muscle mass index; HFrEF, heart failure with reduced ejection fraction; LDL‐C, low‐density lipoprotein cholesterol. Figure S4: Kaplan–Meier survival curves according to ASMI and LDL‐C levels. (A) Statin subgroup. (B) Nonstatin subgroup. ASMI, appendicular skeletal muscle mass index; LDL‐C, low‐density lipoprotein cholesterol. Figure S5: Impact of LDL‐C levels (A) and ASMI (B) on all‐cause mortality in patients with or without statin use. ASMI, appendicular skeletal muscle mass index; LDL‐C, low‐density lipoprotein cholesterol. Figure S6: Impact of LDL‐C levels on all‐cause mortality according to the ASMI value in the nonstatin subgroup. ASMI, appendicular skeletal muscle mass index; LDL‐C, low‐density lipoprotein cholesterol. Figure S7: Kaplan–Meier survival curves according to ASMI and LDL‐C levels (patients surviving > 2 years). ASMI, appendicular skeletal muscle mass index; LDL‐C, low‐density lipoprotein cholesterol. Figure S8: Impact of LDL‐C levels and ASMI on all‐cause mortality (patients surviving > 2 years). ASMI, appendicular skeletal muscle mass index; LDL‐C, low‐density lipoprotein cholesterol. Figure S9: Impact of LDL‐C levels on all‐cause mortality according to the ASMI value (patients surviving > 2 years). A [file JCSM-17-e70168-s001.zip › Supplementary Figure 4(B).TIF]

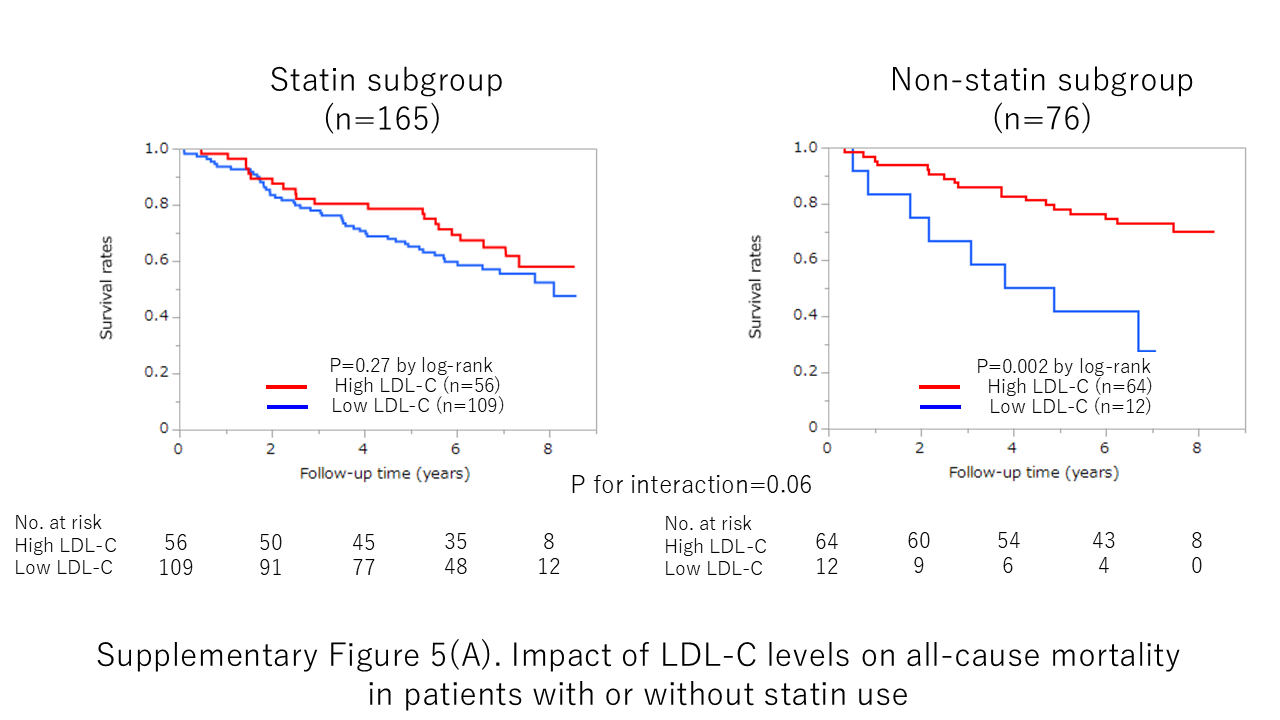

Supplement: Supplementary file 2 — Figure S1: Kaplan–Meier survival curves according to ASMI and LDL‐C levels. (A) HFpEF subgroup. (B) HFrEF subgroup. ASMI, appendicular skeletal muscle mass index; HFpEF, heart failure with preserved ejection fraction; HFrEF, heart failure with reduced ejection fraction; LDL‐C, low‐density lipoprotein cholesterol. Figure S2: Impact of LDL‐C levels (A) and ASMI (B) on all‐cause mortality in patients with HFpEF and HFrEF. ASMI, appendicular skeletal muscle mass index; HFpEF, heart failure with preserved ejection fraction; HFrEF, heart failure with reduced ejection fraction; LDL‐C, low‐density lipoprotein cholesterol. Figure S3: Impact of LDL‐C levels on all‐cause mortality according to the ASMI value in patients with HFrEF. ASMI, appendicular skeletal muscle mass index; HFrEF, heart failure with reduced ejection fraction; LDL‐C, low‐density lipoprotein cholesterol. Figure S4: Kaplan–Meier survival curves according to ASMI and LDL‐C levels. (A) Statin subgroup. (B) Nonstatin subgroup. ASMI, appendicular skeletal muscle mass index; LDL‐C, low‐density lipoprotein cholesterol. Figure S5: Impact of LDL‐C levels (A) and ASMI (B) on all‐cause mortality in patients with or without statin use. ASMI, appendicular skeletal muscle mass index; LDL‐C, low‐density lipoprotein cholesterol. Figure S6: Impact of LDL‐C levels on all‐cause mortality according to the ASMI value in the nonstatin subgroup. ASMI, appendicular skeletal muscle mass index; LDL‐C, low‐density lipoprotein cholesterol. Figure S7: Kaplan–Meier survival curves according to ASMI and LDL‐C levels (patients surviving > 2 years). ASMI, appendicular skeletal muscle mass index; LDL‐C, low‐density lipoprotein cholesterol. Figure S8: Impact of LDL‐C levels and ASMI on all‐cause mortality (patients surviving > 2 years). ASMI, appendicular skeletal muscle mass index; LDL‐C, low‐density lipoprotein cholesterol. Figure S9: Impact of LDL‐C levels on all‐cause mortality according to the ASMI value (patients surviving > 2 years). A [file JCSM-17-e70168-s001.zip › Supplementary Figure 5(A).TIF]

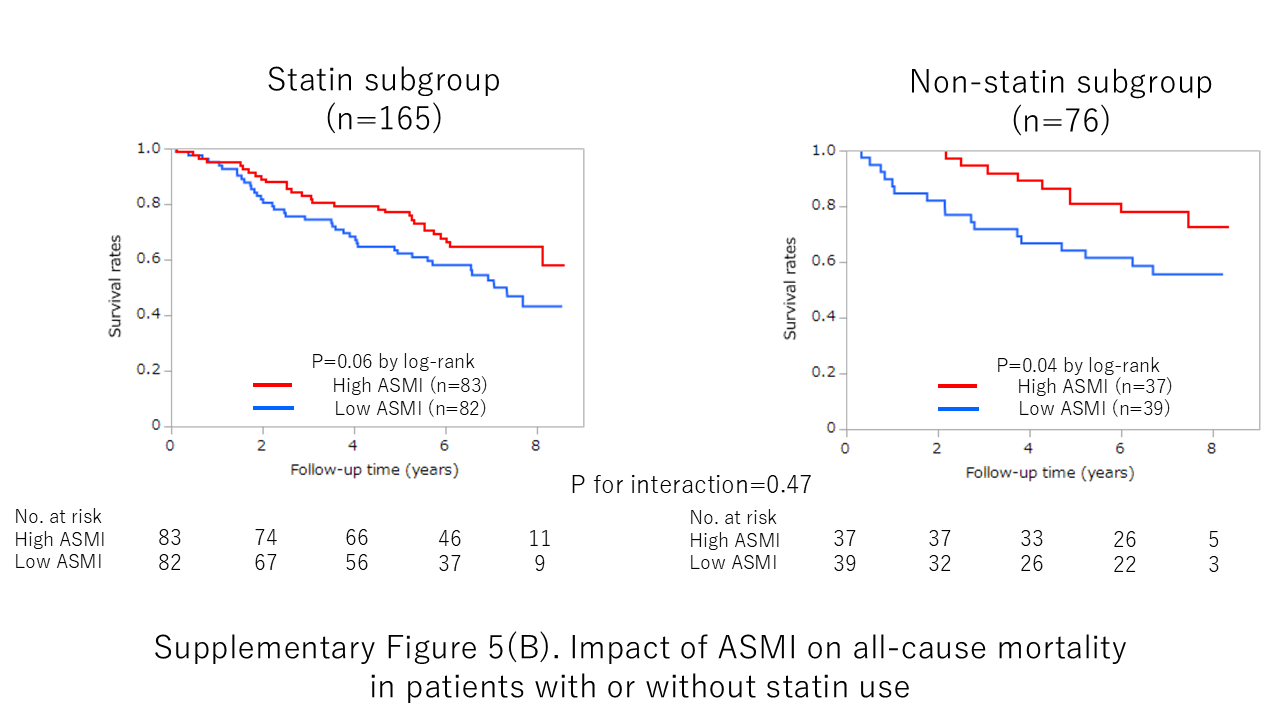

Supplement: Supplementary file 2 — Figure S1: Kaplan–Meier survival curves according to ASMI and LDL‐C levels. (A) HFpEF subgroup. (B) HFrEF subgroup. ASMI, appendicular skeletal muscle mass index; HFpEF, heart failure with preserved ejection fraction; HFrEF, heart failure with reduced ejection fraction; LDL‐C, low‐density lipoprotein cholesterol. Figure S2: Impact of LDL‐C levels (A) and ASMI (B) on all‐cause mortality in patients with HFpEF and HFrEF. ASMI, appendicular skeletal muscle mass index; HFpEF, heart failure with preserved ejection fraction; HFrEF, heart failure with reduced ejection fraction; LDL‐C, low‐density lipoprotein cholesterol. Figure S3: Impact of LDL‐C levels on all‐cause mortality according to the ASMI value in patients with HFrEF. ASMI, appendicular skeletal muscle mass index; HFrEF, heart failure with reduced ejection fraction; LDL‐C, low‐density lipoprotein cholesterol. Figure S4: Kaplan–Meier survival curves according to ASMI and LDL‐C levels. (A) Statin subgroup. (B) Nonstatin subgroup. ASMI, appendicular skeletal muscle mass index; LDL‐C, low‐density lipoprotein cholesterol. Figure S5: Impact of LDL‐C levels (A) and ASMI (B) on all‐cause mortality in patients with or without statin use. ASMI, appendicular skeletal muscle mass index; LDL‐C, low‐density lipoprotein cholesterol. Figure S6: Impact of LDL‐C levels on all‐cause mortality according to the ASMI value in the nonstatin subgroup. ASMI, appendicular skeletal muscle mass index; LDL‐C, low‐density lipoprotein cholesterol. Figure S7: Kaplan–Meier survival curves according to ASMI and LDL‐C levels (patients surviving > 2 years). ASMI, appendicular skeletal muscle mass index; LDL‐C, low‐density lipoprotein cholesterol. Figure S8: Impact of LDL‐C levels and ASMI on all‐cause mortality (patients surviving > 2 years). ASMI, appendicular skeletal muscle mass index; LDL‐C, low‐density lipoprotein cholesterol. Figure S9: Impact of LDL‐C levels on all‐cause mortality according to the ASMI value (patients surviving > 2 years). A [file JCSM-17-e70168-s001.zip › Supplementary Figure 5(B).TIF]

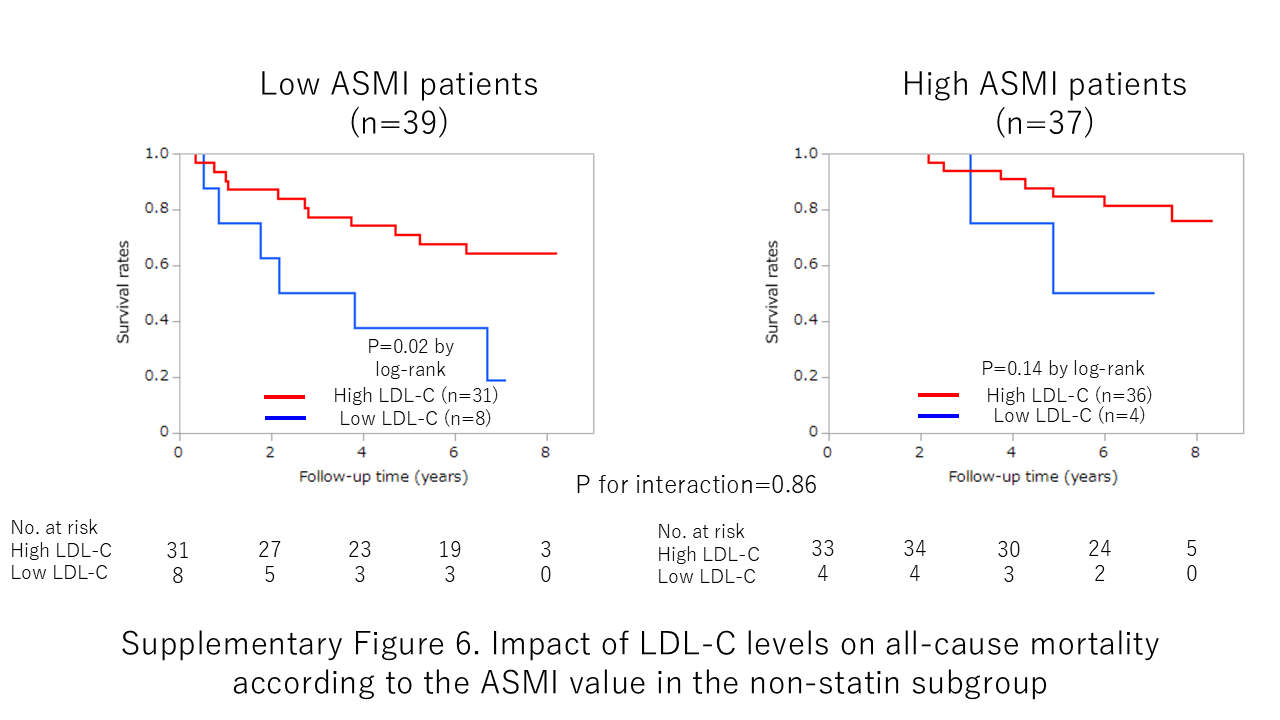

Supplement: Supplementary file 2 — Figure S1: Kaplan–Meier survival curves according to ASMI and LDL‐C levels. (A) HFpEF subgroup. (B) HFrEF subgroup. ASMI, appendicular skeletal muscle mass index; HFpEF, heart failure with preserved ejection fraction; HFrEF, heart failure with reduced ejection fraction; LDL‐C, low‐density lipoprotein cholesterol. Figure S2: Impact of LDL‐C levels (A) and ASMI (B) on all‐cause mortality in patients with HFpEF and HFrEF. ASMI, appendicular skeletal muscle mass index; HFpEF, heart failure with preserved ejection fraction; HFrEF, heart failure with reduced ejection fraction; LDL‐C, low‐density lipoprotein cholesterol. Figure S3: Impact of LDL‐C levels on all‐cause mortality according to the ASMI value in patients with HFrEF. ASMI, appendicular skeletal muscle mass index; HFrEF, heart failure with reduced ejection fraction; LDL‐C, low‐density lipoprotein cholesterol. Figure S4: Kaplan–Meier survival curves according to ASMI and LDL‐C levels. (A) Statin subgroup. (B) Nonstatin subgroup. ASMI, appendicular skeletal muscle mass index; LDL‐C, low‐density lipoprotein cholesterol. Figure S5: Impact of LDL‐C levels (A) and ASMI (B) on all‐cause mortality in patients with or without statin use. ASMI, appendicular skeletal muscle mass index; LDL‐C, low‐density lipoprotein cholesterol. Figure S6: Impact of LDL‐C levels on all‐cause mortality according to the ASMI value in the nonstatin subgroup. ASMI, appendicular skeletal muscle mass index; LDL‐C, low‐density lipoprotein cholesterol. Figure S7: Kaplan–Meier survival curves according to ASMI and LDL‐C levels (patients surviving > 2 years). ASMI, appendicular skeletal muscle mass index; LDL‐C, low‐density lipoprotein cholesterol. Figure S8: Impact of LDL‐C levels and ASMI on all‐cause mortality (patients surviving > 2 years). ASMI, appendicular skeletal muscle mass index; LDL‐C, low‐density lipoprotein cholesterol. Figure S9: Impact of LDL‐C levels on all‐cause mortality according to the ASMI value (patients surviving > 2 years). A [file JCSM-17-e70168-s001.zip › Supplementary Figure 6.TIF]

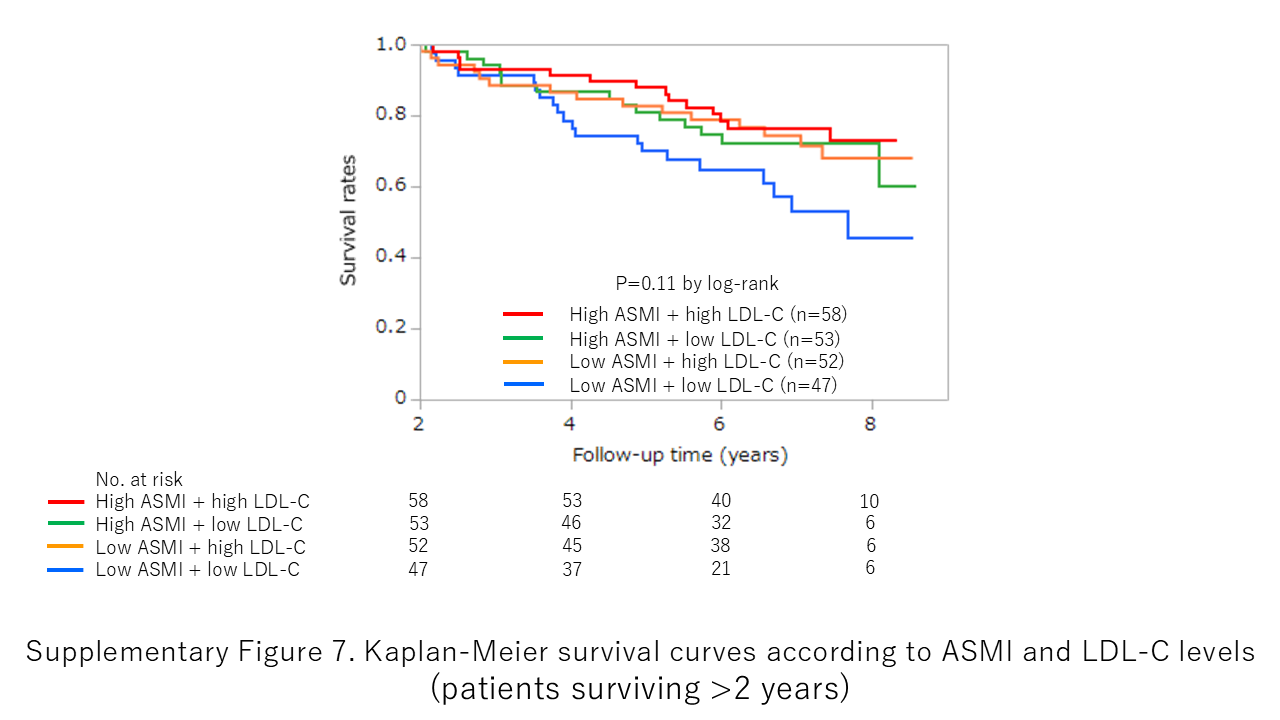

Supplement: Supplementary file 2 — Figure S1: Kaplan–Meier survival curves according to ASMI and LDL‐C levels. (A) HFpEF subgroup. (B) HFrEF subgroup. ASMI, appendicular skeletal muscle mass index; HFpEF, heart failure with preserved ejection fraction; HFrEF, heart failure with reduced ejection fraction; LDL‐C, low‐density lipoprotein cholesterol. Figure S2: Impact of LDL‐C levels (A) and ASMI (B) on all‐cause mortality in patients with HFpEF and HFrEF. ASMI, appendicular skeletal muscle mass index; HFpEF, heart failure with preserved ejection fraction; HFrEF, heart failure with reduced ejection fraction; LDL‐C, low‐density lipoprotein cholesterol. Figure S3: Impact of LDL‐C levels on all‐cause mortality according to the ASMI value in patients with HFrEF. ASMI, appendicular skeletal muscle mass index; HFrEF, heart failure with reduced ejection fraction; LDL‐C, low‐density lipoprotein cholesterol. Figure S4: Kaplan–Meier survival curves according to ASMI and LDL‐C levels. (A) Statin subgroup. (B) Nonstatin subgroup. ASMI, appendicular skeletal muscle mass index; LDL‐C, low‐density lipoprotein cholesterol. Figure S5: Impact of LDL‐C levels (A) and ASMI (B) on all‐cause mortality in patients with or without statin use. ASMI, appendicular skeletal muscle mass index; LDL‐C, low‐density lipoprotein cholesterol. Figure S6: Impact of LDL‐C levels on all‐cause mortality according to the ASMI value in the nonstatin subgroup. ASMI, appendicular skeletal muscle mass index; LDL‐C, low‐density lipoprotein cholesterol. Figure S7: Kaplan–Meier survival curves according to ASMI and LDL‐C levels (patients surviving > 2 years). ASMI, appendicular skeletal muscle mass index; LDL‐C, low‐density lipoprotein cholesterol. Figure S8: Impact of LDL‐C levels and ASMI on all‐cause mortality (patients surviving > 2 years). ASMI, appendicular skeletal muscle mass index; LDL‐C, low‐density lipoprotein cholesterol. Figure S9: Impact of LDL‐C levels on all‐cause mortality according to the ASMI value (patients surviving > 2 years). A [file JCSM-17-e70168-s001.zip › Supplementary Figure 7.TIF]

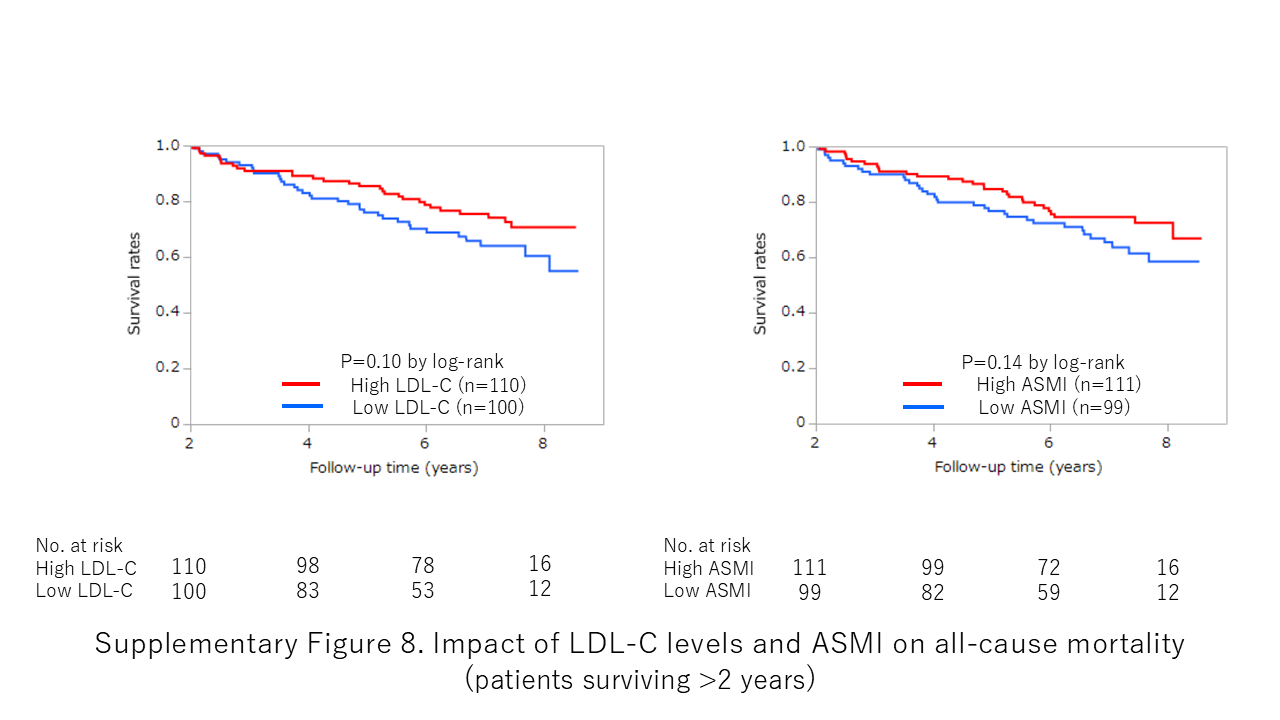

Supplement: Supplementary file 2 — Figure S1: Kaplan–Meier survival curves according to ASMI and LDL‐C levels. (A) HFpEF subgroup. (B) HFrEF subgroup. ASMI, appendicular skeletal muscle mass index; HFpEF, heart failure with preserved ejection fraction; HFrEF, heart failure with reduced ejection fraction; LDL‐C, low‐density lipoprotein cholesterol. Figure S2: Impact of LDL‐C levels (A) and ASMI (B) on all‐cause mortality in patients with HFpEF and HFrEF. ASMI, appendicular skeletal muscle mass index; HFpEF, heart failure with preserved ejection fraction; HFrEF, heart failure with reduced ejection fraction; LDL‐C, low‐density lipoprotein cholesterol. Figure S3: Impact of LDL‐C levels on all‐cause mortality according to the ASMI value in patients with HFrEF. ASMI, appendicular skeletal muscle mass index; HFrEF, heart failure with reduced ejection fraction; LDL‐C, low‐density lipoprotein cholesterol. Figure S4: Kaplan–Meier survival curves according to ASMI and LDL‐C levels. (A) Statin subgroup. (B) Nonstatin subgroup. ASMI, appendicular skeletal muscle mass index; LDL‐C, low‐density lipoprotein cholesterol. Figure S5: Impact of LDL‐C levels (A) and ASMI (B) on all‐cause mortality in patients with or without statin use. ASMI, appendicular skeletal muscle mass index; LDL‐C, low‐density lipoprotein cholesterol. Figure S6: Impact of LDL‐C levels on all‐cause mortality according to the ASMI value in the nonstatin subgroup. ASMI, appendicular skeletal muscle mass index; LDL‐C, low‐density lipoprotein cholesterol. Figure S7: Kaplan–Meier survival curves according to ASMI and LDL‐C levels (patients surviving > 2 years). ASMI, appendicular skeletal muscle mass index; LDL‐C, low‐density lipoprotein cholesterol. Figure S8: Impact of LDL‐C levels and ASMI on all‐cause mortality (patients surviving > 2 years). ASMI, appendicular skeletal muscle mass index; LDL‐C, low‐density lipoprotein cholesterol. Figure S9: Impact of LDL‐C levels on all‐cause mortality according to the ASMI value (patients surviving > 2 years). A [file JCSM-17-e70168-s001.zip › Supplementary Figure 8.TIF]

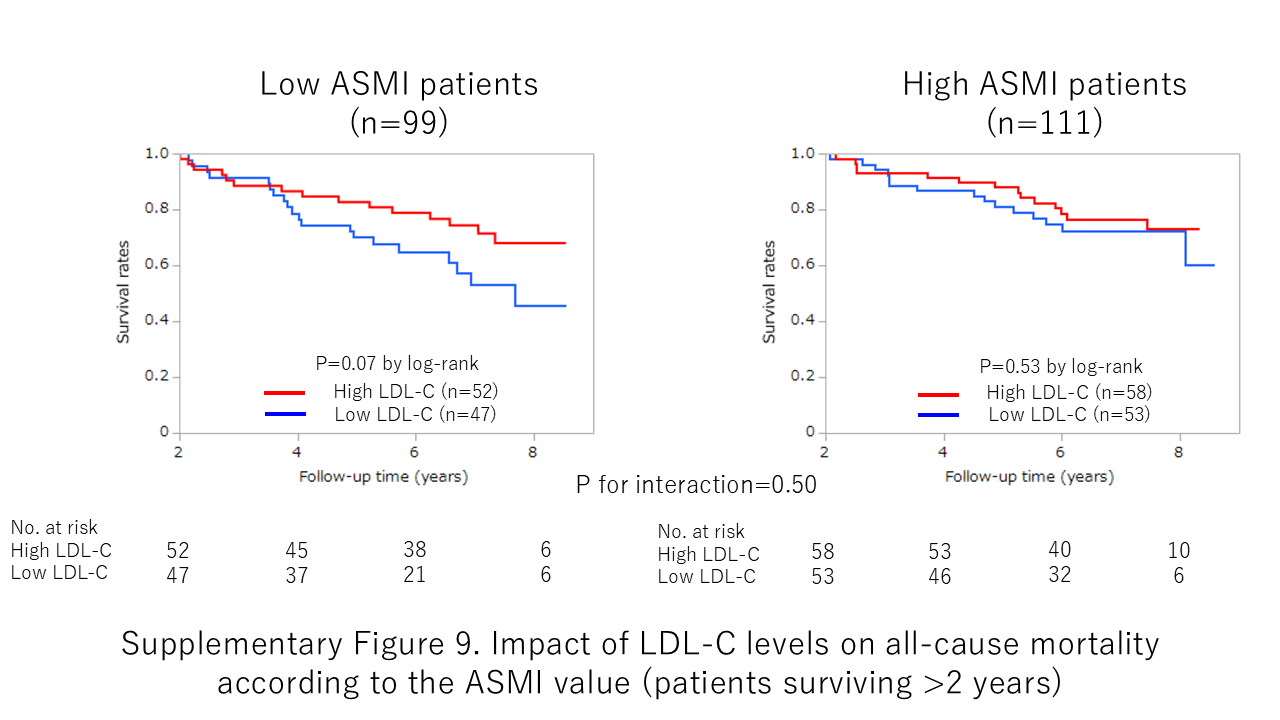

Supplement: Supplementary file 2 — Figure S1: Kaplan–Meier survival curves according to ASMI and LDL‐C levels. (A) HFpEF subgroup. (B) HFrEF subgroup. ASMI, appendicular skeletal muscle mass index; HFpEF, heart failure with preserved ejection fraction; HFrEF, heart failure with reduced ejection fraction; LDL‐C, low‐density lipoprotein cholesterol. Figure S2: Impact of LDL‐C levels (A) and ASMI (B) on all‐cause mortality in patients with HFpEF and HFrEF. ASMI, appendicular skeletal muscle mass index; HFpEF, heart failure with preserved ejection fraction; HFrEF, heart failure with reduced ejection fraction; LDL‐C, low‐density lipoprotein cholesterol. Figure S3: Impact of LDL‐C levels on all‐cause mortality according to the ASMI value in patients with HFrEF. ASMI, appendicular skeletal muscle mass index; HFrEF, heart failure with reduced ejection fraction; LDL‐C, low‐density lipoprotein cholesterol. Figure S4: Kaplan–Meier survival curves according to ASMI and LDL‐C levels. (A) Statin subgroup. (B) Nonstatin subgroup. ASMI, appendicular skeletal muscle mass index; LDL‐C, low‐density lipoprotein cholesterol. Figure S5: Impact of LDL‐C levels (A) and ASMI (B) on all‐cause mortality in patients with or without statin use. ASMI, appendicular skeletal muscle mass index; LDL‐C, low‐density lipoprotein cholesterol. Figure S6: Impact of LDL‐C levels on all‐cause mortality according to the ASMI value in the nonstatin subgroup. ASMI, appendicular skeletal muscle mass index; LDL‐C, low‐density lipoprotein cholesterol. Figure S7: Kaplan–Meier survival curves according to ASMI and LDL‐C levels (patients surviving > 2 years). ASMI, appendicular skeletal muscle mass index; LDL‐C, low‐density lipoprotein cholesterol. Figure S8: Impact of LDL‐C levels and ASMI on all‐cause mortality (patients surviving > 2 years). ASMI, appendicular skeletal muscle mass index; LDL‐C, low‐density lipoprotein cholesterol. Figure S9: Impact of LDL‐C levels on all‐cause mortality according to the ASMI value (patients surviving > 2 years). A [file JCSM-17-e70168-s001.zip › Supplementary Figure 9.TIF]
