# Supplementary material for: Skeletal Muscle Mass Modifies the Prognostic Impact of LDL Cholesterol in Chronic Heart Failure
Source: J Cachexia Sarcopenia Muscle. 2026 Jan 16;17(1):e70168. doi: 10.1002/jcsm.70168 (PMC12809719; doi:10.1002/jcsm.70168)
Supplement: Supplementary file 3 — Data S1: Supporting Information. [file JCSM-17-e70168-s002.docx]

STROBE Statement—checklist of items that should be included in reports of observational studies

|  | | | Item No. | Recommendation | Page  No. | | | Relevant text from manuscript |
| --- | --- | --- | --- | --- | --- | --- | --- | --- |
| **Title and abstract** | | | 1 | (*a*) Indicate the study’s design with a commonly used term in the title or the abstract | 1,3-5 | | | **Title**" and "**Abstract**" section |
|  |  |  |  | (*b*) Provide in the abstract an informative and balanced summary of what was done and what was found | 3-5 | | | "**Abstract**" section |
| Introduction | | | | | | | |  |
| Background/rationale | | | 2 | Explain the scientific background and rationale for the investigation being reported | 6,7 | | | "Dyslipidemia is one of the major risk factors…whether skeletal muscle mass modifies the association between LDL-C levels and prognosis in patients with HF." |
| Objectives | | | 3 | State specific objectives, including any prespecified hypotheses | 7 | | | "the present study aims to investigate the association between LDL-C levels and all-cause mortality in patients with chronic HF in the context of skeletal muscle mass.^21,22^" |
| Methods | | | | | | | |  |
| Study design | | | 4 | Present key elements of study design early in the paper | 8,9 | | | "**Study population**" section |
| Setting | | | 5 | Describe the setting, locations, and relevant dates, including periods of recruitment, exposure, follow-up, and data collection | 8,9 | | | "**Study population**" section |
| Participants | | | 6 | (*a*) *Cohort study*—Give the eligibility criteria, and the sources and methods of selection of participants. Describe methods of follow-up  *Case-control study*—Give the eligibility criteria, and the sources and methods of case ascertainment and control selection. Give the rationale for the choice of cases and controls  *Cross-sectional study*—Give the eligibility criteria, and the sources and methods of selection of participants | 8-10 | | | "**Study population**" and  "**Clinical follow-up**" section |
|  |  |  |  | (*b*) *Cohort study*—For matched studies, give matching criteria and number of exposed and unexposed  *Case-control study*—For matched studies, give matching criteria and the number of controls per case | Not applicable | | |  |
| Variables | | | 7 | Clearly define all outcomes, exposures, predictors, potential confounders, and effect modifiers. Give diagnostic criteria, if applicable | 7,9,10 | | | "the present study aims to investigate the association between LDL-C levels and all-cause mortality in patients with chronic HF in the context of skeletal muscle mass.^21,22^"  "Appendicular skeletal muscle mass index (ASMI) was calculated…an appendicular skeletal muscle mass two standard deviations below the mean of a healthy young reference group aged 18 to 40 (men <7.26 kg/m2, women <5.45 kg/m2).^23^"  "Based on the median values (93 mg/dL), patients were divided into low and high LDL-C groups." |
| Data sources/ measurement | | | 8 | For each variable of interest, give sources of data and details of methods of assessment (measurement). Describe comparability of assessment methods if there is more than one group | 9.10 | | | "**Assessment of muscle mass, fat mass, muscle strength, and exercise capacity**" section  "**Laboratory analysis**" section |
| Bias | | | 9 | Describe any efforts to address potential sources of bias | 11,12 | | | "Independent variables for model development were selected based on clinical relevance and the results of the univariate analysis… and repeated the survival analyses to confirm whether the observed associations persisted thereafter." |
| Study size | | | 10 | Explain how the study size was arrived at | 8 | | | "From March 2010 to April 2012, we initially enrolled 329 subjects in the SICA-HF project at Charité – Universitätsmedizin Berlin, Campus Virchow-Klinikum, Germany. After excluding controls and subjects missing survival data, lipid profiles, or body composition measurements, a total of 241 ambulatory HF patients with complete data (survival data, lipid profiles, and dual-energy X-ray absorptiometry (DEXA) scans) were retrospectively analyzed in this substudy (**Figure 1**)." |
| Quantitative variables | | 11 | | Explain how quantitative variables were handled in the analyses. If applicable, describe which groupings were chosen and why | 9,10,11 | | "Appendicular skeletal muscle mass index (ASMI) was calculated…an appendicular skeletal muscle mass two standard deviations below the mean of a healthy young reference group aged 18 to 40 (men <7.26 kg/m2, women <5.45 kg/m2).^23^"  "Based on the median values (93 mg/dL), patients were divided into low and high LDL-C groups."  "**Statistical analysis**" section | |
| Statistical methods | | 12 | | (*a*) Describe all statistical methods, including those used to control for confounding | 10-12 | | "**Statistical analysis**" section | |
|  |  |  |  | (*b*) Describe any methods used to examine subgroups and interactions | 11,12 | | "To further validate the robustness of the findings, we conducted several subgroup and sensitivity analyses… differed by HF phenotypes and statin use." | |
|  |  |  |  | (*c*) Explain how missing data were addressed | 12 | | "As missing data were minimal and deemed unlikely to affect the results substantially, no imputation was applied and all analyses were based on complete cases." | |
|  |  |  |  | (*d*) *Cohort study*—If applicable, explain how loss to follow-up was addressed  *Case-control study*—If applicable, explain how matching of cases and controls was addressed  *Cross-sectional study*—If applicable, describe analytical methods taking account of sampling strategy | 10 | | No patient in the analyzed subset (n=241) was lost to follow-up. | |
|  |  |  |  | (*e*) Describe any sensitivity analyses | 11 | | "Finally, as an additional sensitivity analysis, we restricted our cohort to patients who survived at least two years from baseline and repeated the survival analyses to confirm whether the observed associations persisted thereafter." | |
| Results | | | | | | | | |
| Participants | | 13 | | (a) Report numbers of individuals at each stage of study—eg numbers potentially eligible, examined for eligibility, confirmed eligible, included in the study, completing follow-up, and analysed | 8 | | "**Study population**" section and  **Figure 1** | |
|  |  |  |  | (b) Give reasons for non-participation at each stage | 8 | | **Figure 1** | |
|  |  |  |  | (c) Consider use of a flow diagram | 8 | | **Figure 1** | |
| Descriptive data | | 14 | | (a) Give characteristics of study participants (eg demographic, clinical, social) and information on exposures and potential confounders | 13 | | **Table 1** | |
|  |  |  |  | (b) Indicate number of participants with missing data for each variable of interest | 8 | | **Figure 1** | |
|  |  |  |  | (c) *Cohort study*—Summarise follow-up time (eg, average and total amount) | 13 | | "During a median follow-up of 6.3 [4.0-7.3] years, 95 patients (39%) died." | |
| Outcome data | | 15 | | *Cohort study*—Report numbers of outcome events or summary measures over time | 13 | | "During a median follow-up of 6.3 [4.0-7.3] years, 95 patients (39%) died." | |
|  |  |  |  | *Case-control study—*Report numbers in each exposure category, or summary measures of exposure |  | |  | |
|  |  |  |  | *Cross-sectional study—*Report numbers of outcome events or summary measures |  | |  | |
| Main results | | 16 | | (*a*) Give unadjusted estimates and, if applicable, confounder-adjusted estimates and their precision (eg, 95% confidence interval). Make clear which confounders were adjusted for and why they were included | 14,15 | | **Figure 2,3, and 5**  **Table 2 and Table 3** | |
|  |  |  |  | (*b*) Report category boundaries when continuous variables were categorized | 11,14 | | "Additionally, to explore the dose-response relationship between LDL-C levels and all-cause mortality, we categorized LDL-C levels into sextiles…"  **Figure 4** | |
|  |  |  |  | (*c*) If relevant, consider translating estimates of relative risk into absolute risk for a meaningful time period | Not applicable | |  | |
| Other analyses | 17 | | Report other analyses done—eg analyses of subgroups and interactions, and sensitivity analyses | | 16-18 | "**Subgroup and sensitivity analyses**" section  **Supplementary Figure 1-9** | | |
| Discussion | | | | | | | | |
| Key results | 18 | | Summarise key results with reference to study objectives | | 19 | "Our study is the first to demonstrate an association…only in patients with low LDL-C levels and low ASMI." | | |
| Limitations | 19 | | Discuss limitations of the study, taking into account sources of potential bias or imprecision. Discuss both direction and magnitude of any potential bias | | 24,25 | "This study has several limitations. First…adipose tissue in HF patients with low LDL-C levels and low ASMI." | | |
| Interpretation | 20 | | Give a cautious overall interpretation of results considering objectives, limitations, multiplicity of analyses, results from similar studies, and other relevant evidence | | 24 | "First, the present study involved a small sample size of patients…these findings should be interpreted cautiously." | | |
| Generalisability | 21 | | Discuss the generalisability (external validity) of the study results | | 21,24 | "In patients with more severe HF, such as those with acute, decompensated, or advanced stages, the clinical impact of low LDL-C levels may be more pronounced. Taken together, further debate is required to determine the optimal lipid management strategy for patients with HF.^37^"  "However, whether the adverse impact of low LDL-C levels depends on HF aetiology or pathophysiology remains unclear, and further investigation is warranted." | | |
| Other information | | |  | | | | | |
| Funding | 22 | | Give the source of funding and the role of the funders for the present study and, if applicable, for the original study on which the present article is based | | 26,27 | "The project was supported by the European Commission’s 7th Framework programme (FP7/2003-2013) under grant agreement number 241558; the Russian Ministry of Science and Education within the FTP ‘R&D in priority fields of the S&T complex of Russia 2007–2012’ under state contract number 02.527.11.0007." | | |
